# Population-Specific Regulation of Chmp2b by *Lbx1* during Onset of Synaptogenesis in Lateral Association Interneurons

**DOI:** 10.1371/journal.pone.0048573

**Published:** 2012-12-21

**Authors:** Jun Xu, Mariko Nonogaki, Ravi Madhira, Hsiao-Yen Ma, Ola Hermanson, Chrissa Kioussi, Michael K. Gross

**Affiliations:** 1 Department of Pharmaceutical Sciences, Oregon State University, Corvallis, Oregon, United States of America; 2 Department of Neuroscience, Karolinska Institutet, Stockholm, Sweden; University of Edinburgh, United Kingdom

## Abstract

Chmp2b is closely related to Vps2, a key component of the yeast protein complex that creates the intralumenal vesicles of multivesicular bodies. Dominant negative mutations in Chmp2b cause autophagosome accumulation and neurodegenerative disease. Loss of Chmp2b causes failure of dendritic spine maturation in cultured neurons. The homeobox gene *Lbx1* plays an essential role in specifying postmitotic dorsal interneuron populations during late pattern formation in the neural tube. We have discovered that Chmp2b is one of the most highly regulated cell-autonomous targets of *Lbx1* in the embryonic mouse neural tube. Chmp2b was expressed and depended on *Lbx1* in only two of the five nascent, Lbx1-expressing, postmitotic, dorsal interneuron populations. It was also expressed in neural tube cell populations that lacked Lbx1 protein. The observed population-specific expression of Chmp2b indicated that only certain population-specific combinations of sequence specific transcription factors allow Chmp2b expression. The cell populations that expressed Chmp2b corresponded, in time and location, to neurons that make the first synapses of the spinal cord. Chmp2b protein was transported into neurites within the motor- and association-neuropils, where the first synapses are known to form between E11.5 and E12.5 in mouse neural tubes. Selective, developmentally-specified gene expression of Chmp2b may therefore be used to endow particular neuronal populations with the ability to mature dendritic spines. Such a mechanism could explain how mammalian embryos reproducibly establish the disynaptic cutaneous reflex only between particular cell populations.

## Introduction

Chmp2b mutations have been observed in Danish [1], Belgian [2], Dutch [3], American [4], and French [5] patients with frontotemporal dementia (FTD). Chmp2b mutations have also been observed in ALS patients [6], [7]. These mutations appear to act in a dominant negative fashion by debilitating autophagosome fusion to lysosomes [8].

Chmp2b and Chmp2a are the two mammalian orthologs of yeast Vps2, a key component of the endosomal sorting complexes required for transport (ESCRT) system. The ESCRT-0, -I, -II, and -III complexes are essential for moving ubiquitinated transmembrane proteins into intralumenal vesicles (ILVs) as endosomes mature into multivesicular bodies (MVBs) [9], [10], [11], [12]. MVBs either fuse with lysosomes to degrade the transmembrane proteins or they fuse with the cell membrane to release the ILVs as exosomes. The ESCRT system creates membrane vesicles that bud away from the cytosol. The biochemical mechanism of budding-away differs fundamentally from the clathrin-dependent mechanism of budding-into the cytosol. Budding-away is used during ILV, autophagosome and exosome formation, as well as for cytokinesis, and viral budding. The ESCRT-0, -I, and -II complexes sequester ubiquitinated cargo and nucleate a nascent ILV bud. The ESCRT-III proteins assemble into a tightening spiral complex at the neck of the bud to pinch it off. None of the early ESCRT III steps are thought to be ATP-dependent, yet the subsequent dissociation of the spiral ESCRT-III complex is. The Vps2 protein occupies a central position in the spiral complex at the end of its assembly and attaches, by a C-terminal domain, to the Vps4 ATPase that drives complex dissociation at the expense of ATP. Vps2 is therefore well positioned to control the rate of ESCRT complex function [13], [14], [15], [16], [17], [18].

Several lines of evidence indicate that Chmp2b is involved in dendritic spine maturation, while Chmp2a plays the role of Vps2 in the late endosomes, multivesicular bodies, and autophagosomes of mammals. First, the Chmp2a protein is more closely related to Vps2 than Chmp2b at the protein sequence level. Second, two-hybrid interaction screens reveal strong physical interactions between Chmp2a and both mammalian Vps4 ATPases, whereas Chmp2b fails to interact [19], [20]. The Chmp2b C-terminal domain forms a far weaker interaction with Vps4 MIT domains than the Chmp2a C-terminal domain [21], [22]. Third, loss of Chmp2b function in cultured cortical [23] or hippocampal [24] neurons failed to produce the defects in vesicular trafficking, autophagosome accumulation, or neuronal death observed with the dominant negative mutations, mentioned above, that cause genetic disease. Instead, the knock-down of Chmp2b levels leads to a reduced ability to mature dendritic spines to form the mushroom shape that is characteristically found in potentiated synapses [24]. Finally, the immunohistochemical puncta observed with normal Chmp2b in hippocampal neurons do not colocalize with early or late endosome markers [24].

Neuronal specification occurs in the embryonic neural tube of mice between embryonic day (E) 9.5 and E13, prior to the elaboration of neuron-specific functions. After dividing cells of the ventricular zone withdraw from the cell cycle, their daughter cells migrate to the mantle zone. Cells at different spatial positions in the dividing ventricular layer express distinct combinations of sequence specific DNA binding transcription factors (SSTFs). The postmitotic daughter neurons that emerge laterally from each spatial domain express new, distinct combinations of SSTFs. Population partitioning analysis has been used to estimate that at 30–45% of the SSTFs in the mammalian genome have spatially constrained expression patterns in the neural tube [25]. A combinational code of SSTF expression is used to define populations of neurons along both the dorsal–ventral and anterior-posterior axes in both the ventricular and mantle zones [26], [27], [28]. These SSTFs are typically linked in cross-regulatory interactions with each other, so that specific combinations of SSTF are likely to form transcriptional network kernels that specify neural tube cell types [29].

Lbx1 is a homeodomain SSTF that is expressed in postmitotic neurons of the dorsal, embryonic neural tube [30], [31]. *Lbx1* is essential to the specification of five dorsal interneuron populations, namely dI4, dI5, dI6, dI4L^A^ and dI4L^B^. Each of these populations expresses a unique, yet only partially known, combination of SSTFs. It has been demonstrated that *Lbx1* alters RNA expression levels of 8% of the genome's 1700 SSTF genes in these five interneuron populations [29]. *Lbx1* regulates SSTF target genes in a population-specific manner. For example, *Lbx1* activates Pax2 and represses Foxd3 expression in dI4, dI6 and dI4L^A^ cells, but not in dI5, or dI4L^B^ cells. Conversely, *Lbx1* activates Lmx1b and represses Isl1 expression in dI5 and dI4L^B^ cells, but not in dI4, dI6 or dI4L^A^ cells. Therefore, the 8% of SSTF genes that are identified as *Lbx1* genetic targets are not targets in each of the five Lbx1-expressing populations. Instead, *Lbx1* regulates a different set of SSTF targets in each population where it is expressed. It is therefore likely that Lbx1 acts as a patterning SSTF by contributing Boolean input to the *cis*-regulatory modules (CRMs) of target genes [29].

We demonstrate in this report that *Lbx1* regulates many non-SSTF target genes in the embryonic neural tube in a cell-autonomous manner and provide a detailed analysis of one of these target genes. The second most strongly regulated non-SSTF target gene was Chmp2b, which is required for dendrite maturation in cultured neurons. *Lbx1*-dependent Chmp2b gene expression was only observed in a specific populations of nascent postmitotic neurons in the embryonic mouse neural tube. Chmp2b protein was observed in the lateral white matter, where the earliest known synapses form in the cervical/thoracic embryonic mouse neural tube at E11.5. These early synapses are known to create the disynaptic spinal reflex, which involves association and motor neurons. Similarly, Chmp2b protein was observed in the dorsolateral funiculus at E12.5, when the first synaptic interactions between the central and peripheral nervous systems are known to occur at this axial level. Genetic control of Chmp2b expression may therefore be used to endow particular embryonic neuronal populations with the ability to stabilize or potentiate excitatory synapses at specific times in development. Overall, the results suggest a molecular mechanism by which developmental gene expression programs could insure that only the appropriate cell types contribute productively to the emergent spinal reflexes and somatosensory circuits.

## Materials and Methods

Immunohistochemistry, flow sorting, microarray sample preparation, microarray processing, microarray analysis, and qPCR confirmation procedures, as well another subset of the data of the indicated arrays have been described previously [25], [30]. For the present report we developed a new *in situ* hybridization procedure for mouse embryos that uses RNA probes prepared with biotinylated–UTP.

### Antibody Characterization

Commercially available primary antibodies against Chmp2b (Abcam 33174); Isl1/2 (40.2d6, Developmental Studies Hybridoma Bank); Lhx1/5 (4F2; Developmental Studies Hybridoma Bank), Brn3a (Eric Turner), Lmx1b (Tom Jessell), ß-tubulin class III isoform (clone TU-20; Mab 1637; Millipore; Tuj1-like), MAP2a (clone AP20; MAB3418 Millipore), and Nestin (DSHB rat-401) were used. The primary antibody against GFP was affinity purified from the serum of rats injected with a His6-tagged full length EGFP protein that had been purified from bacteria. The specificity and background staining of this antibody was established by comparing titrations on sections from Lbx1^GFP/+^ and ICR embryos. Cy2, Cy3, and Cy5 labeled donkey-anti-IgG secondary antibodies that had been preabsorbed against extensive panels of IgGs of other species were obtained from Jackson Immunoresearch.

### Mouse Embryo Preparation

The Institutional Animal Care and Use Committee at Oregon State University approved all animal procedures. The Lbx1^GFP^ allele [32] has been maintained in outbred ICR mice by continuous outcrossing with ICR males purchased from the Jackson Laboratories for over 20 generations. The day of vaginal plug was considered E0.5. Embryos were dissected from the uterus in ice-cold phosphate buffered saline (1×PBS; 2.7 mM KCl, 1.37 M NaCl, 10 mM Na_2_HPO_4_ and 1.7 mM KH_2_PO_4_ adjusted to pH7.4) and genotyped by inspection under a fluorescent dissection microscope. Lbx1^+/+^ mice lack green fluorescence, Lbx1^GFP/+^ embryos have fluorescent neural tubes and limb buds, and Lbx1^GFP/GFP^ embryos have fluorescent neural tubes but non-fluorescent limb buds. Embryos were fixed in 4% PF (1 part 20% paraformaldehyde in 1×PBS, 1 part 1M sodium phosphate (pH 7.4), 3 parts water) at 4°C. Fixation was typically 0.5 h for immunohistochemistry and 48 h for in situ hybridization. Embryos were cryoprotected for 24 hours each in 10% and 25% sucrose at 4°C. Pairs, or triplets, of stage-matched littermates of different genotypes were aligned and embedded together in single blocks of OCT (Sakura, Ltd) in a dry ice/ethanol bath. This insured that all subsequent processing conditions were identical between the genotypes to be compared. Blocks were stored at −70°C in air-tight, dessicated containers until cryostat sections of 16–20 µm were cut and lifted onto Superfrost Plus slides (VWR). Slides air-dried on a warm block (25°C) for several hours prior to desiccated, frozen storage (−70°C) or direct use.

### Preparation of Biotinylated RNA Probes

PCR generated template (PGT) for *in vitro* transcription was produced by amplifying the cDNA insert of pYX-Chmp2b (MGC: 67644; IMAGE clone: 6414077) using T7 (TAATACGACTCACTATAGGG) and T3 (AATTAACCCTCACTAAAGGG) primers. Similar PGTs were created for full-length protein coding sequences of mouse Pax2, mouse Lmx1b, and EGFP. Antisense RNA probes were prepared from 100 ng PGT in a 20 µl reaction containing 40 mM Tris pH7.9, 10 mM NaCl, 6 mM MgCl_2_, 2 mM spermidine, 1 mM DTT, 20 U T3 RNA Polymerase (Promega), 40 U RNase OUT (Fermentas), 0.35 mM biotinylated–UTP (biotin-16-UTP-Li_4_; 987.5 g/mol; Epicentre), 0.65 mM UTP, 1 mM ATP, 1 mM GTP and 1 mM CTP (Bioline). Reactions were incubated at 37°C for 6 hours. Additional T3 polymerase (1 µl, 20 U) was added and incubation continued overnight. Probes were purified on calibrated G50 gel filtration columns. A 5 ml disposable plastic pipette was cut at the top, fitted with a frit of baked glass wool, and filled to the 5 ml mark with Sephadex G50 (fine), swollen in DEPC-H_2_O. Reactions were layered onto the surface of the matrix and DEPC-H_2_O was applied. Elution fractions were taken at 0.5 ml intervals and their absorbance at 260 nm was measured on aliquots using a Nanodrop spectrophotometer. The first peak to be eluted was used as the RNA probe. Absorbance returned to baseline before a second peak, presumably containing unincorporated nucleotides, was observed. Virtually all unincorporated biotinylated-UTP was therefore removed from probes. Less rigorous purification methods such as spin columns yielded probe preparations that produced high levels of nonspecific signal in hybridizations. Approximately 20 µg of probe were typically recovered.

### In situ Hybridization with Biotinylated Probes

All reagents used prior to the first wash step were prepared with water, 20×SSC (3 M NaCl, 0.3 M Sodium Citrate and adjusted pH to 7.0 with HCl), 1 M Na PO_4_, or 1×PBS that had been stirred vigorously with 0.1%(v/v) diethylpyrocarbonate (DEPC) overnight and autoclaved for 2 h. Formamide (Sigma) was deionized by stirring with 100 g/L mixed-bed ion exchange resin (IONAC NM-60 H^+^/OH^−^ form type I beads;16–50 mesh; J. T. Baker) for 24 h. Resin was removed by Büchner filtration and aliquots were frozen.

Slides were post-fixed in a humidified chamber for 30 min in 4% PF at room temperature (RT), washed three times for five minutes (3×5′) with 1×PBS at RT, transferred to 5-slot slide mailers (12.5 ml with 5 slides; Healthrow Scientific) and pre-hybridized for 2 h at 68°C in hybridization buffer (50% deionized formamide, 5×SSC, 50 µg/ml yeast tRNA (Boehringer Mannheim), 50 µg/ml Heparin (Sigma), 50 mM NaPO_4_ pH 4.0, 0.1% SDS). Probes were denatured 15 min at 75°C, snap-chilled on ice, added to 68°C hybridization buffer at 0.5 ng/µl, and applied to the slides in parafilm-sealed slide mailers at 68°C for 16 h. Slides were quickly, and individually, rinsed by dunking into prewarmed (68°C) Wash Buffer (50% deionized formamide, 5×SSC and 1% SDS) and placed into prewarmed (68°C) 2×SSC for 20 min in a fresh slide mailer. Slides were washed once more with prewarmed (68°C) 2×SSC for 20 min, treated with 0.05 µg/ml RNase in prewarmed (37°C) RNase buffer (0.5 M NaCl, 10 mM Tris-HCl pH 7.5, 1 mM EDTA) for 5 min, rinsed with RNase Buffer (lacking RNAse) at RT, washed 3×5′ with RNase Buffer (lacking RNAse) at RT, washed 3×5′ with prewarmed (68°C) 0.2×SSC, and washed 3×5′ with TBST (TBS (25 mM Tris pH 7.5, 137 mM NaCl, 5 mM KCl, 0.5 mM MgCl_2_) containing 0.1% Tween 20) at RT. Washes were done by exchanging fluids in each mailer and gently rocking in a water bath.

Slides were placed on racks in a humidified chamber and Blocking Solution (4% nonfat dry milk in TBS) was applied for 30 min at RT. Streptavidin conjugated alkaline phosphatase (SA-AP; 1 mg/ml; Jackson Immunoresearch) was diluted 1∶500 in Blocking Solution and applied overnight at RT. Slides were washed 3×20′ with TBST at RT and transferred to slide mailers containing Color Reaction Buffer (0.1 M NaCl, 50 mM MgCl_2_, 10 mM Tris-HCl pH 9.5, 0.1% Tween and 2 mM levamisole) at RT for 10 min. Slides were incubated at 37°C in Color Reaction Buffer containing 0.33 mg/ml nitro-blue tetrazolium chloride (NBT; CAS 298-83-9; Amresco) and 0.165 mg/ml 5-bromo-4-chloro-3′-indolyphosphate p-toluidine salt (BCIP; CAS 6578-06-9; Amresco). Frozen NBT and BCIP stocks were prepared at 5% (w/v) in 70% and 100% dimethylformamide, respectively. Color reactions were stopped with PBS when signal to noise ratios were judged to be optimal. Slides were rinsed briefly with water, dehydrated using an alcohol series (5 min each in 50%, 70%, 95%, 100% ethanol) followed by xylene (2×5′) that was kept anhydrous by Molecular Sieves (Sigma), and mounted with DPX (VWR).

### Image Acquisition and Processing

NBT/BCIP colored in situ images were recorded with a Micropublisher 5.0 (QImaging) camera, which contains a CCD chip with a 3-color Bayer filter. Images were converted to grey scale in Adobe Photoshop and then assigned false colors using red, blue, or green color tables. False color images were combined and aligned as layers in Adobe Photoshop.

Immunofluorescence images were recorded on a Zeiss Axioplan Microscope using a 16 bit Zeiss Axiocam monochrome camera. Monochrome images taken with appropriate filter sets for Cy2, Cy3, or Cy5 were imported into the RGB color channels of Adobe Photoshop at full spatial resolution. Exposure times were manually fixed (automation off) for each color during the recording of images across experiments to insure that identical exposures were taken of sections that were to be directly compared. Only a subset of the 16-bit dynamic range acquired by Axiocam contains relevant image data. Automation selects a subset of the range for display and export based on the individual image, rather than across an experiment. Automation was therefore turned off and the subset of the 16 bit dynamic range that was exported to Adobe Photoshop was manually fixed across experiments for each color. This portion of the acquired dynamic range was scaled to 256 levels within each color channel of Adobe Photoshop (8 bit color). Level adjustment layers were applied to individual images in the figure layouts to further refine the dynamic range selection of each color channel for optimal representation. These level adjustments were similar, but not identical, across experiments. Brightness, contrast, and gamma values were never altered. Images shown in this report are therefore direct linear representations of recorded image intensities. Comparison shown between genotypes represent identical sample processing and image acquisition times, and have only very minor differences in the selection of dynamic range. Channel Mixer Adjustment layers were used to rotate the colors of images for optimal representation, as indicated in the figure legends.

## Results

### Activation of Chmp2b Expression by Lbx1

**Figure 1:**
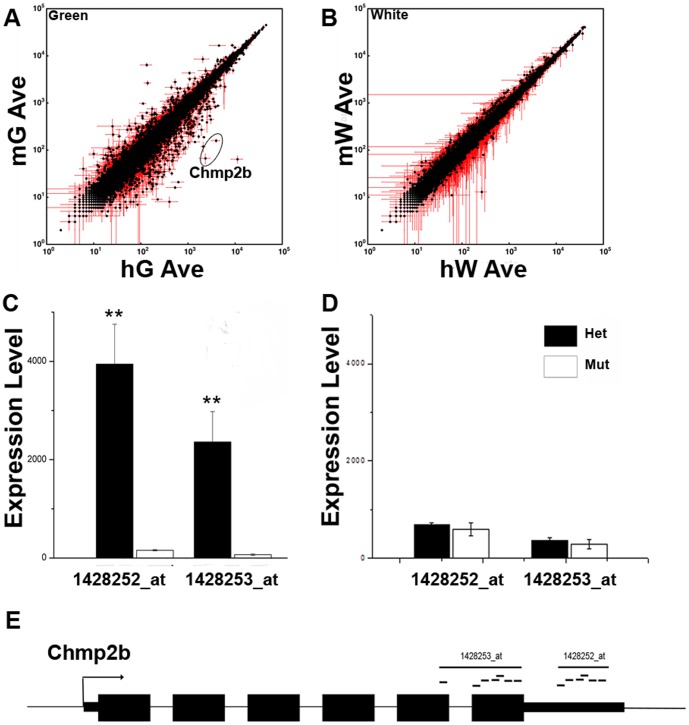
Non-SSTF, Cell-Autonomous, Neural Tube, Genetic Target Genes. Neural tubes were dissected from heterozygote Lbx1^GFP/+^(h) and mutant Lbx1^GFP/GFP^ (m) E12.5 embryos. Pools of neural tubes from each genotype were rapidly dissociated, flow sorted into GFP^+^ (green; G) and GFP^−^ (white; W) cells, and total RNA prepared. Three replicates of the flow sort experiment were used to produce triplicate array data for each of the four conditions, namely heterozygous green (hG), mutant green (mG), heterozygous white (hW), and mutant white (mW). Error bars in each direction indicate the standard deviation in three biological replicates. Only 41,500 of the array's probe sets, corresponding to 22,300 non-sequence specific transcription factor (non-SSTF) genes are shown. (A) Cell autonomous changes in gene expression due to loss of *Lbx1* function were measured in cells that normally express Lbx1 (green cells). Chmp2b probe sets are circled. Significant (P<0.05; *t*-test) changes in 3400 probes sets, corresponding to 2200 genes were observed. Similar numbers of probe sets had significantly higher (1660) or lower (1730) signals in mG, respectively. At least 1000 and 1200 genes were genetically repressed and activated by *Lbx1*, respectively. (B) Non-cell autonomous changes in gene expression due to loss of *Lbx1* function were measured in cells that normally lack Lbx1 expression (white cells). Significant (P<0.05; *t*-test) changes in 1600 probe sets, corresponding to 930 genes were observed. Similar numbers of probe sets had significantly higher (880) or lower (690) signals in mW, respectively. At least 550 and 700 genes were genetically repressed and activated by *Lbx1*, respectively. The magnitude of cell autonomous changes (shown in A) was generally far greater than the magnitude of non-cell autonomous changes (shown in B). (C–E) Array data for two different Chmp2b probe sets (E) shown as bar graphs for green cells (C) and white cells (D).

The enhanced green fluorescent protein (GFP) coding sequence has been knocked-in to the Lbx1 locus to create the *Lbx1^GFP^* null allele, which expresses GFP instead of Lbx1 protein [32]. Neural tubes were dissected from heterozygous (Lbx1^GFP/+^) and mutant (Lbx1^GFP/GFP^) embryos at E12.5. Dissected neural tubes were dissociated and sorted into GFP^+^ (green) and GFP^−^ (white) cell types as previously described [25]. RNA from these cells was used to probe Affymetrix U430 2.0 microarrays. Three biological replicates were prepared for each of the four conditions (hG, heterozygous green; mG, mutant green; hW, heterozygous white; mW, mutant white). The average signal of the three replicates was used to identify *Lbx1*-dependent changes in expression of non-SSTF genes (Fig. 1 A, B). There were far more *Lbx1*-dependent changes in green cells than white cells. White cells normally lack Lbx1 expression. Consequently, the loss of both *Lbx1* alleles would not be expected to cause cell-autonomous changes in gene expression in white cells. In contrast, green cells normally express Lbx1 protein and the loss of the final functional *Lbx1* allele in these cells leads to a large number of significant changes in gene expression in non-SSTF genes. In this report we will focus on only one of these genetic target genes, namely Chmp2b.

Two of the largest *Lbx1*-dependent fold-changes were observed with the two Chmp2b probe sets (Fig. 1A, circled). These probe sets were directed against the 3′ nontranslated region and last two exons of Chmp2b (Fig. 1E), and showed 25- and 35- fold higher signals in green cells of heterozygotes than in green cells of mutants (Fig. 1C). Thus, *Lbx1* function was required for Chmp2b expression in green cells. Heterozygous white cells expressed 6-fold less Chmp2b mRNA than heterozygous green cells (compare black bars in Fig. 1C, D). However, no *Lbx1*-dependent difference in Chmp2b expression was observed in white cells (compare black and white bars in Fig. 1D). Chmp2b expression was therefore *Lbx1*-independent in cells lacking Lbx1 expression.

### Lbx1-Dependent and -Independent Chmp2b Expression Domains

**Figure 2:**
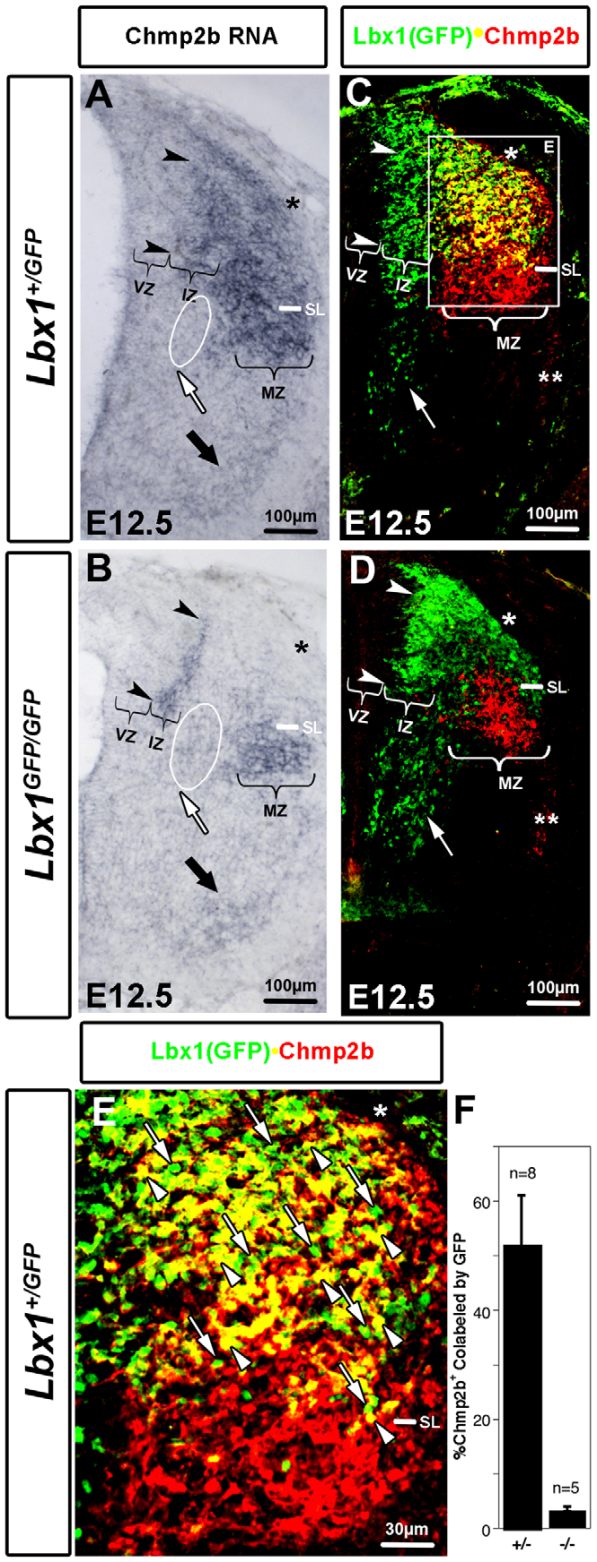
Lbx1-Dependent and Lbx1-Independent Chmp2b Expression Domains. (A,B) Chmp2b RNA at mid-forelimb levels in the dorsal and ventral portions of the mantle zone is *Lbx1*-dependent and *Lbx1*-independent, respectively. RNA expression in the motor columns (black arrow) varied considerably in adjacent sections, but was not generally affected when series of sections along corresponding parts of the body axis were compared. Arrowheads indicate the region of the intermediate zone (IZ), where Chmp2b RNA signal remains intense in mutants. The IZ lies lateral and adjacent to the ventricular zone (VZ). Chmp2b signal can be compared in the circumferential trajectory at the level of the sulcus limitans (SL), in the circled region (white arrow). (C, D) Chmp2b protein at E12.5 at mid-forelimb levels in the dorsal and ventral portions of the mantle zone (MZ) is Lbx1-dependent and *Lbx1*-independent, respectively. The dorsolateral funiculus (DLF; *) and ventrolateral funiculus (VLF; **) are indicated. (E) Only a subset of the Lbx(GFP)^+^ cells within the lateral dorsal horn express Chmp2b (arrowheads). Green cells lacking Chmp2b protein are intermingled (arrows). (F) Quantitative evaluation of the fraction of Chmp2b^+^ area in the neural tube that was colabeled by GFP. All images of the neural tube in this report will be oriented medial to lateral (left to right) and dorsal to ventral (top to bottom).

The green cells of E12.5 neural tubes are postmitotic, occupy most of the dorsal half of the neural tube, and give rise to the substantia gelatinosa and other cell types of the adult dorsal horn. A biotinylated probe directed against the entire Chmp2b open reading frame (ORF) was used to detect Chmp2b RNA expression in neural tube cross sections of heterozygote and mutant embryos at E12.5 (Fig. 2). Several sets of serial sections along the body axis from pairs of embryos were analyzed. Representative images at forelimb levels are shown. Chmp2b RNA was observed in heterozygotes, within the dorsal region where the flow-sorted green cells normally reside, as well as in more ventral regions of the mantle zone (Fig. 2A). Chmp2b RNA expression in mutants was not observed in the dorsal region, but was still observed in the more ventral region of the mantle zone (Fig. 2B). The selective loss of Chmp2b expression above the *sulcus limitans* strikingly confirmed the loss of expression observed in green cells in the microarray analysis. The retention of signal below the sulcus limitans confirmed the *Lbx1*-independent expression in white cells in the microarray analysis.

The loss of Chmp2b RNA was not complete. Three residual expression domains remained in mutants. One robust domain was located in the mantle zone immediately below the *sulcus limitans* and another weak domain was located in the ventral horn near the motor neurons (black arrow). Both of these were clearly outside of the normal Lbx1 expression domain, and therefore represented *Lbx1*-independent domains of Chmp2b expression. The third residual expression domain of mutants was observed as a medial column, at the position where Lbx1^+^ neurons normally emerge from the ventricular zone (between arrowheads). Based on Nomarski viewing, this intense column of RNA expression appeared to be more closely associated with the ventricular zone, and more dense, than the medial edge of the heterozygote expression domain.. Its presence in mutants suggests that Chmp2b RNA expression was initiated but not maintained in some of the populations that normally express Lbx1.

Double-labeling immunohistochemistry confirmed the RNA analyses at the protein level (Fig. 2C,D). In heterozygotes, the GFP antibody labeled both newborn neurons in an intermediate zone, adjacent to the ventricular zone, and more mature neurons in the lateral mantle zone. The anti-Chmp2b antibody only labeled cells in the lateral mantle zone of heterozygotes. The ventral half of the Chmp2b expression domain did not overlap with the GFP expression domain and was not lost in mutants. It was therefore *Lbx1*-independent. The dorsal half of the Chmp2b expression domain overlapped with the GFP expression domain and was absent in mutant embryos. It was therefore *Lbx1*-dependent (Fig. 2D). Approximately half (52%) of the Chmp2b^+^ surface in heterozygotes was colabeled with GFP. This dropped to a 3% in mutants (Fig. 2F), indicating a complete loss of Chmp2b in the GFP^+^ cell populations. No GFP^+^Chmp2b^+^ cells could be confirmed in mutants.

Chmp2b protein was not detected medially in the GFP expression domain of heterozygotes, suggesting that Lbx1 protein appears prior to Chmp2b protein in dorsal interneurons. Chmp2b protein was also not observed in the medial column of mutants where intense Chmp2b RNA had been observed, indicating that the RNA was not translated into sufficient Chmp2b protein to detect. Very low levels of Chmp2b protein were detected in motor neurons and along the circumferential trajectory in subsequent analyses.

Lbx1-expressing neuronal populations are respecified in *Lbx1* mutants. They begin to resemble more dorsal populations. They alter their normal lateral migration route and move, instead, along the circumferential trajectory associated with commissural neurons [30]. GFP^+^ cells become more numerous along the circumferential trajectory of mutants (Fig. 2 C,D,white arrows). Very little Chmp2b RNA expression was observed along this trajectory in heterozygotes (Fig. 2A, white arrow/circled area), indicating that Chmp2b RNA was not expressed in the dI6 population. This was confirmed by immunohistochemistry (Fig. 2C).

Some Chmp2b RNA was observed in the circumferential trajectory of mutants (Fig. 2B; white arrow/circled area), but did not persist to the floor plate. Chmp2b protein was also not observed in the circumferential trajectory of mutants (Fig. 2 D; white arrows), again consistent with the idea that respecified cells initiate Chmp2b RNA expression, but do not maintain it or produce protein from it.

### Chmp2b Expression Present in dI4L^A^ and Not Detected in dI4L^B^

Chmp2b protein signal in the dorsolateral neural tube above the *sulcus limitans* was associated with only a subset of the GFP^+^ neurons (Fig. 2E). Approximately half of the cells in this region were yellow (Lbx1(GFP)^+^Chmp2b^+^; arrowheads) and half were green (Lbx1(GFP)^+^Chmp2b^−^; arrows). This observation indicates that Chmp2b was expressed in only a subset of the Lbx1-expressing dorsolateral cells of heterozygotes.

The selective expression of Chmp2b in only a subset of Lbx1(GFP)^+^ cells of the nascent dorsal horn was interesting because the five Lbx1 expressing populations are anatomically intermingled. A spatial signaling cue from an exterior anatomical structure that effects selective Chmp2b activation, would be expected to lead to graded, rather than intermingled, Chmp2b expression. Local signaling from afferent axons to individual cells in the horn is not possible because primary afferents have not entered the dorsal gray matter at this stage of development [33]. A final possibility is that population-specific Chmp2b expression is brought about by cell-intrinsic mechanisms like the ones that control many SSTF expression patterns during neuronal specification.

It is well established that two distinct, Lbx1^+^ populations born from the dI4 progenitor layer between E11.75 and E13 colonize this region. These populations, referred to as dI4L^A^ and dI4L^B^, are distinguished by their mutually exclusive expression of Pax2 and Lmx1b, respectively. Unlike the three earlier-born Lbx1^+^ populations (dI4, dI5, and dI6), they are not born in dorso-ventrally segregated groups. Instead, they are born in an intermingled fashion. After birth they move laterally to mix with the earlier born populations.

**Figure 3:**
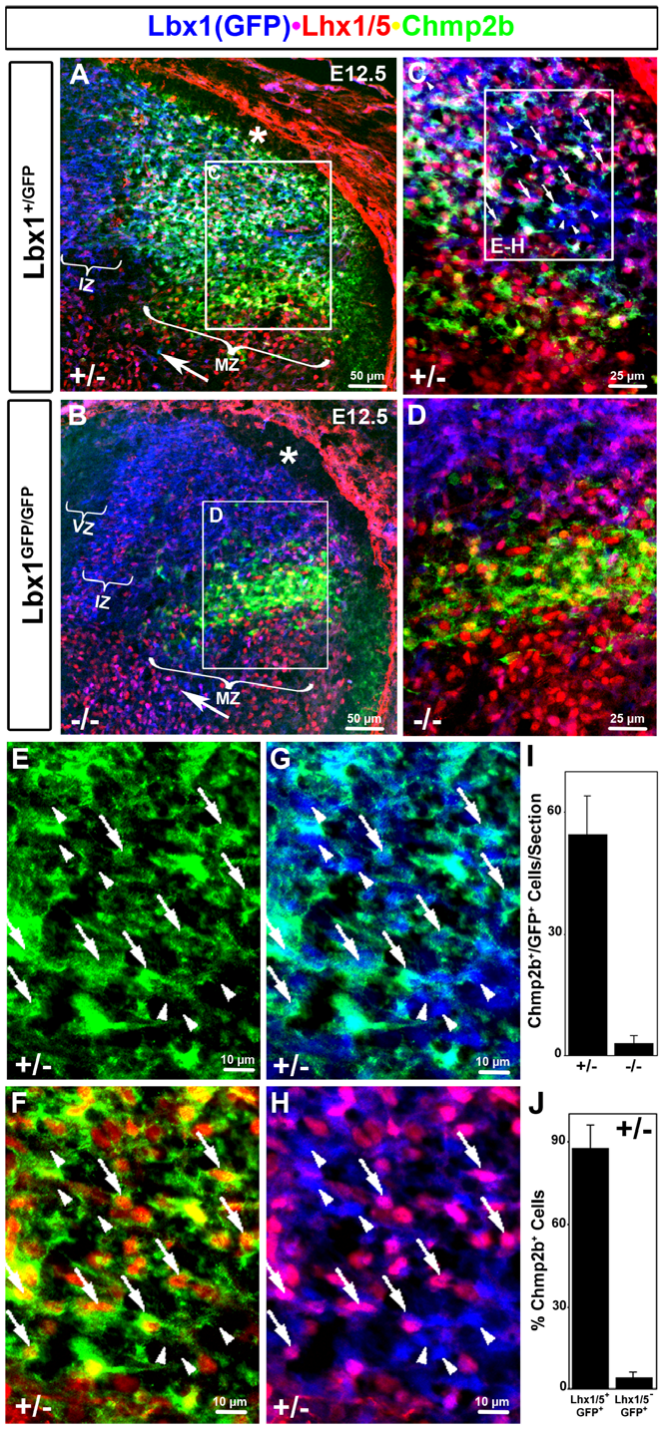
Population-Specific Chmp2b in dI4L^A^. (A, B) Low magnification overviews of heterozygote and mutant E12.5 dorsal horn at mid-forelimb levels. Note both the loss of Chmp2b and the severe reduction of Lhx1/5 in the dorsal horn mantle of mutants below the DLF (*). Note retention of Chmp2b in a more ventral GFP^−^ zone below the sulcus limitans. Supernumerary GFP^+^ cells are seen in circumferential trajectory (white arrows) ventral to this zone. (C, D) Insets shown in A and B. (E–H) Magnified images of the triple labeled inset shown in C. This region contains spatially intermingled dI4L^A^ (GFP^+^Lhx1/5^+^) and dI4L^B^ (GFP^+^Lhx1/5^−^) cells. Dual and single channel images of the same field are used to illustrate the selective expression of Chmp2b in dI4L^A^ cells. The arrows or arrowheads indicate the same two sets of cells in all four panels. Arrows indicate GFP stained cells that contain Lhx1/5 stained nuclei. Chmp2b signal tightly surrounds these nuclei. Arrowheads indicate GFP stained cells that lack Lhx1/5 stained nuclei. Chmp2b staining is not associated with these cells. Primary antibodies against GFP (rat), Lhx1/5 (mouse), and Chmp2b (rabbit) were detected using appropriate Cy2 (green), Cy3 (red), and Cy5 (infrared) secondary antibodies, respectively. Colors in the images were switched for clarity to those indicated by the labels.(I, J) Blind quantification of cells in GFP/Lhx1/5/Chmp2b stained sections of heterozygote (n = 8) and mutant (n = 6). VZ, ventricular zone; IZ, intermediate zone; MZ, mantle zone; SL, sulcus limitans; DLF (*), dorsolateral funiculus.

**Figure 4:**
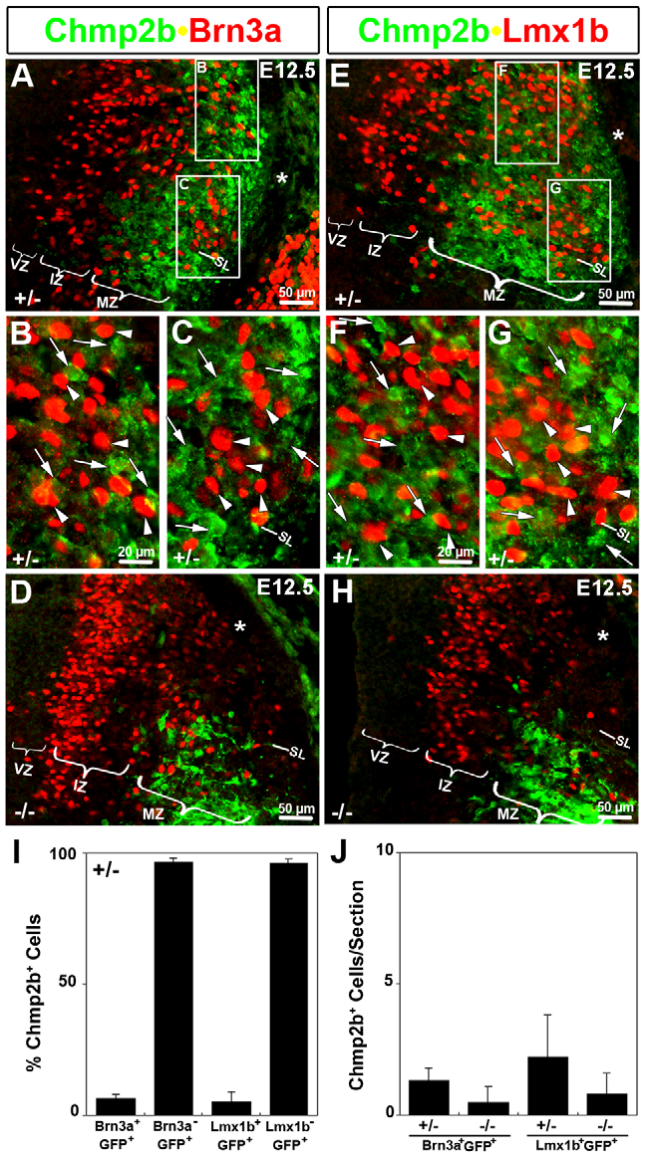
Lack of Chmp2b in dI4L^B^ Late Population. Lmx1b and Brn 3a mark both the dI4L^B^ and dI5 populations of interneurons at E12.5 in mid-forelimb cross sections. These populations express Lbx1(GFP) (not shown), but lack Chmp2b. (A–C) Nuclei stained with the Brn3a antibody are located within the region stained by Chmp2b antibodies. Dorsal and ventral clusters of these nuclei correspond to the dI4L^B^ and dI5 populations, respectively. Brn3a stained nuclei (arrowheads) in these regions are not associated with Chmp2b staining. Other cell bodies in the immediate vicinity lack Brn3a and are stained for Chmp2b (arrows). (D) Loss of Chmp2b and severe reduction of Brn3a in dorsal horn of mutants. (E–G) Nuclei stained with the Lmx1b antibody are located within the region stained by Chmp2b antibodies. Dorsal and ventral clusters of these nuclei correspond to the dI4L^B^ and dI5 populations, respectively. Lmx1b stained nuclei (arrowheads) in these regions are not associated with Chmp2b staining. Other cell bodies in the immediate vicinity lack Lmx1b and are stained for Chmp2b (arrows). (H) Loss of Chmp2b and severe reduction of Lmx1b in dorsal horn of mutants. Primary antibodies against GFP (rat), Brn3a (guinea pig) or Lmx1b (guinea pig), and Chmp2b (rabbit) were detected using appropriate Cy2(green), Cy3(red), and Cy5 (infrared) secondary antibodies. The Lbx1(GFP) channel was omitted and the colors of the remaining channels switched for clarity to those indicated by the labels. (I, J) Blind quantification of cells in Brn3a/GFP/Chmp2b stained sections for heterozygote (n = 7) and mutants (n = 4), and Lmx1b/GFP/Chmp2b stained sections for heterozygote (n = 5) and mutants (n = 6). VZ, ventricular zone; IZ, intermediate zone; MZ, mantle zone; SL, sulcus limitans; DLF (*), dorsolateral funiculus.

Co-labeling immunohistochemistry of anti-Chmp2b antibody together with various combinations of antibodies against GFP, Pax2, Lmx1b, Brn3a, and Lhx1/5 at E12.5 (Figs. 3, 4) was used to ask if Chmp2b expression was restricted to specific Lbx1^+^ cell populations. Pax2 or Lhx1/5 antibodies are typically used, in conjunction with an Lbx1 antibody, to identify the dI4L^A^, dI4, and dI6 populations. A direct comparison of Pax2 and Chmp2b was precluded because both available antibodies are derived in rabbits. However, triple labeling with GFP, Chmp2b, and Lhx1/5 antibodies revealed that Chmp2b expression was within the dI4L^A^ population (Fig. 3). Lhx1/5 staining was largely confined to the lateral mantle zone region and therefore marks more mature cells. High magnification triple stained images of the dorsal horn were photographed and the color channels were dissociated into pairs to allow the cytoplasmic Chmp2b, whole-cell GFP, and nuclear Lhx1/5 immunoreativities to be more readily compared (Fig. 3 E–H). Chmp2b immunoreactivity (Fig. 3E) surrounded the Lhx1/5^+^ Lbx1(GFP)^+^ nuclei in the dorsal horn (arrows). In contrast, intermingled Lhx1/5^−^ Lbx1(GFP)^+^ nuclei were not associated with Chmp2b staining (arrowheads). The Chmp2b staining associated with Lhx1/5^+^ Lbx1(GFP)^+^ nuclei in the dorsal horn was not detected in this region in mutants (Fig. 3 A vs. B, C vs. D). Consequently, the *Lbx1*-dependent expression domain of Chmp2b is composed mainly, or entirely, of dI4 and/or dI4L^A^ cells.

Cells were marked as circles in the relevant area of the dorsal horn using the GFP and Lhx1/5 staining channels in the absence of Chmp2b signal. Cells were then blindly scored for Lhx1/5^+^GFP^+^ (Chmp2b channel off), Lhx1/5^−^GFP^+^ (Chmp2b channel off), Chmp2b^+^(Lhx1/5, GFP channels off), and Chmp2b^−^ (Lhx1/5, GFP channels off). The results from eight dorsal horn images indicate that 90% of the Lhx1/5^+^GFP^+^ cells are associated with Chmp2b signal, whereas only 9% of the Lhx1/5^−^GFP^+^ cell are associated with Chmp2b signal (Fig. 3 J). The large percentage of Lhx1/5^+^GFP^+^ cells that express Chmp2b at E12.5 precludes the idea that these are merely early born dI4 cells. A similar analysis in mutants was irrelevant because no Chmp2b+GFP+ cells can be confirmed (Fig. 3I).

The Lmx1b and Brn3a antibodies, which mark both the dI4L^B^ and dI5 populations, stain nuclei in both the medial intermediate zone and the lateral mantle zone regions of the dorsal horn at E12.5 (Fig. 4 A, E). The nuclei of the medial intermediate zone are adjacent to the ventricular zone, in a region containing predominantly newborn neurons. Although Chmp2b RNA was readily observed in this medial zone (Fig. 2A), robust Chmp2b protein expression was typically only detected laterally (Fig. 4A,E). Low levels of Chmp2b protein were sometimes observed in the intermediate zone in particularly well-developed stains. Some Brn3a^+^ or Lmx1b^+^ nuclei were embedded in the Chmp2b stained dorsolateral region that contains more mature dI4L^B^ cells (Fig. 4 A, E and top insets B, F) and in a lateral cell cluster near the sulcus limitans that contains dI5 cells (Fig. 4 A, E and bottom insets C, G). High magnification insets show that the Brn3a^+^ or Lmx1b^+^ nuclei are not associated with cytoplasmic Chmp2b immunoreactivity (Fig. 4 B, C, F, G). Lhx1/5^+^ nuclei neatly fill nuclear-shaped holes in the Chmp2b stain (Fig. 3), while Lmx1b^+^ and Brn3a^+^ nuclei do not (Fig. 4).

GFP^+^ cells were marked in the relevant area of micrographs in the absence of Chmp2b signal. The relevant area did not include the Chmp2b^−^ GFP^+^ medial area. Marked cells were blindly scored for Brn3a (Chmp2b channel off), Lmx1b(Chmp2b channel off), or Chmp2b (Brn3a, Lmx1b, GFP channels off). Only 5% of the Brn3a^+^GFP^+^ or Lmx1b^+^GFP^+^ cells were associated with Chmp2b signal (Fig. 4I). Few, if any Brn3a^+^GFP^+^ or Lmx1b^+^GFP^+^ cells were detected in heterozygotes or mutants (Fig. 4J). In contrast, 95% of the Brn3a^−^GFP^+^ or Lmx1b^−^GFP^+^ in th relevant area were associated with Chmp2b signal (Fig. 4I). Taken together the data show that Chmp2b was selectively expressed in Lbx1^+^ dI4/dI4L^A^ cells but not in the intermingled Lbx1^+^ dI5/dI4L^B^ cells.

### dI4-Specific Expression of Chmp2b in Early-Born Lbx1^+^ Cells

**Figure 5:**
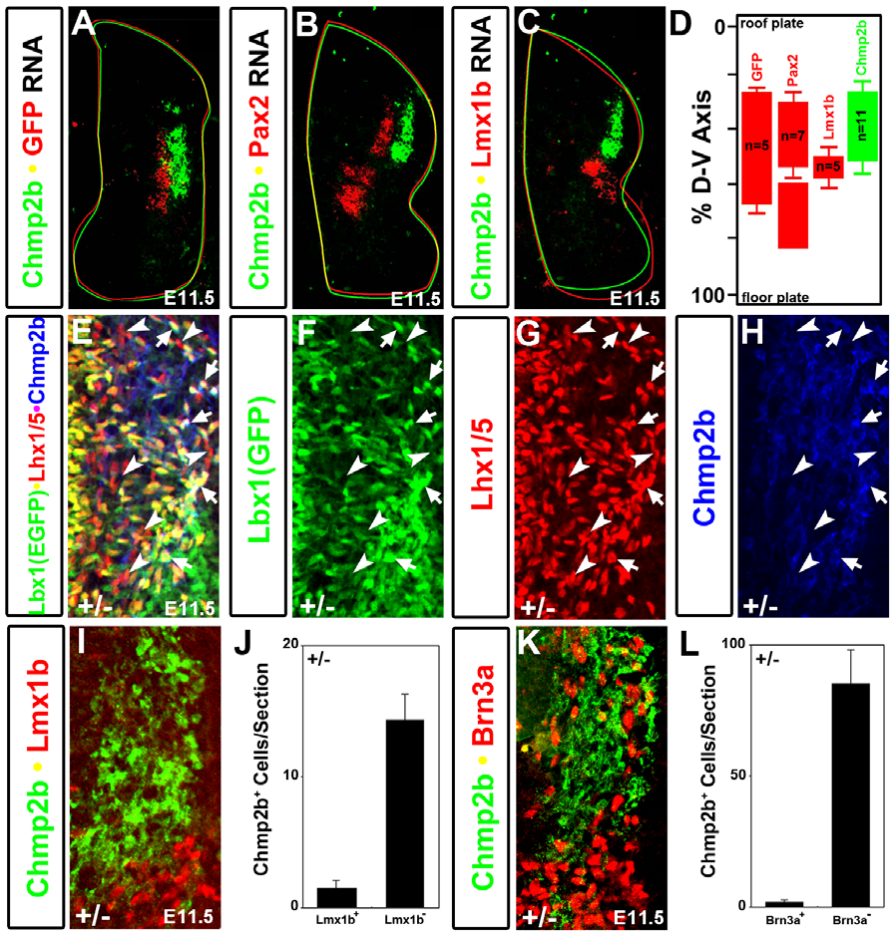
Population-specific Expression at Early Stage. Lbx1 expressing cells emerge as a single column from the ventricular zone from E10.5 to E11.75. They belong to three populations, named dI4, dI5, and dI6, adjacent to each other along the dorsal-ventral axis. (A–C) *In situ* hybridizations with the indicated probes were done on adjacent E11.5 sections of heterozygotes at mid-forelimb level. Signals were photographed in color, converted to greyscale, and rendered false color using color tables. The contours of the basal lamina and lumen were traced and used to align images. Chmp2b RNA is observed adjacent to the dorsal portion of the Lbx1(GFP) RNA column (A) where Pax2 RNA (B) but not Lmx1b RNA (C) is expressed. (D) Summary of parallel section analyses along the body axis. (E–H) A single triple-labeled image (E) is shown in single color channels (F–H). Arrows and arrowheads indicate the same two sets of cells in these four images. Arrows indicate dI4 cells that co-label with the Lbx1(GFP) and Lhx1/5 antibodies. Chmp2b antibody stains these cells. They are either within the lateral column or horizontally-oriented and appear to be moving toward the lateral column. Arrowheads indicate cells dI2 cells that stain with Lhx1/5 but not Lbx1(GFP). These cells are vertically oriented and are not stained by the Chmp2b antibody. (I) Cells of the dI5 populations are labeled by Lmx1b, but not by Chmp2b, antibodies. Single channel images of this image are shown in Fig. S1. (J) Quantification of cells in Chmp2b/Lmx1b/GFP stained heterozygote sections (n = 4). (K) Cells of the dI1, dI2, dI3, and dI5 populations are labeled by Brn3a, but not by Chmp2b, antibodies. Single channel images of this image are shown in Fig. S1. (L) Quantification of cells in Chmp2b/Brn3a/GFP stained heterozygote sections (n = 4). Signals for Lhx1/5, Brn3a, and Lmx1b were developed with Cy3(red) secondary antibodies. Signals for Lbx1(GFP) and Chmp2b were developed with Cy2 (green) and Cy5 (infrared) secondary antibodies, respectively. Channels were omitted or colors were switched, for clarity, to those indicated by the labels.

Lbx1 is also expressed in the earlier-born dI4, dI5, and dI6 populations. These three early populations are best observed at E10.5 at forelimb levels. Chmp2b RNA or protein expression was not observed anywhere in the neural tube at this stage (data not shown). Chmp2b RNA was first observed at E11.5 in a lateral column at all axial levels of the spinal cord (Fig. 5 A–D). The two late populations, dI4L^A^ and dI4L^B^, are not yet born at E11.5. Chmp2b RNA was therefore expressed in at least a subset of the three early populations. Its expression domain resembled the known Lbx1 expression domain.

In situ hybridizations were performed on adjacent sections using probes against Chmp2b, GFP, Pax2, or Lmx1b (Fig. 5, A–D). Purple color from the color reactions were rendered in false color using red or green color tables in Adobe Photoshop. The basal lamina and lumen were traced and adjacent sections were aligned by these traces to determine the relative positions of the RNA signals. Clearly, Chmp2b RNA was expressed lateral to the Lbx1(GFP) and Pax2 RNAs (Fig. 5A, B), but dorsal to the Lmx1b RNA (Fig. 5C). The column of Chmp2b RNA was lateral only to dorsal portions of the Pax2 and Lbx1(GFP) columns. Quantitative evaluations of dorso-ventral extent of each of the RNAs expression domains (Fig. 5D) suggested that Chmp2b was expressed in the dI4, but not the dI5 or dI6 populations.

Immunohistochemical experiments confirmed this interpretation. Lhx1/5 expression marks both the dI2 and dI4 populations at E11.5. The dI2 population is commissural, located along the circumferential trajectory, and lacks Lbx1. The dI4 population is located in a lateral column, and expresses Lbx1. Triple labeling with Chmp2b, GFP, and Lhx1/5 antibodies can therefore be used to identify the dI4 population positively (Fig. 5 E–H). Chmp2b protein was observed in Lbx1(GFP)^+^Lhx1/5^+^ cells (arrows) in the lateral column, but was not observed in Lbx1(GFP)^−^Lhx1/5^+^ cells (arrowheads) along the circumferential trajectory. Thus, Chmp2b was expressed in dI4, but not dI2 cells.

Lmx1b is expressed only in the dI5 neurons at E11.5. No co-labeling was observed with Lmx1b and Chmp2b antibodies (Fig. 5I, J; single channel images of Fig. 5I are shown in Fig. S1). Chmp2b was also not observed in cells in the circumferential trajectory ventral to the Lmx1b expression domain, and was therefore not expressed in dI6 cells (Data not shown). Although the three genetically-specified, early, Lbx1^+^ populations (dI4, dI5, and dI6) are spatially adjacent at E11.5, only the dI4 population expressed Chmp2b.

### Chmp2b Expression in a Subset of the dI1 Population

**Figure 6:**
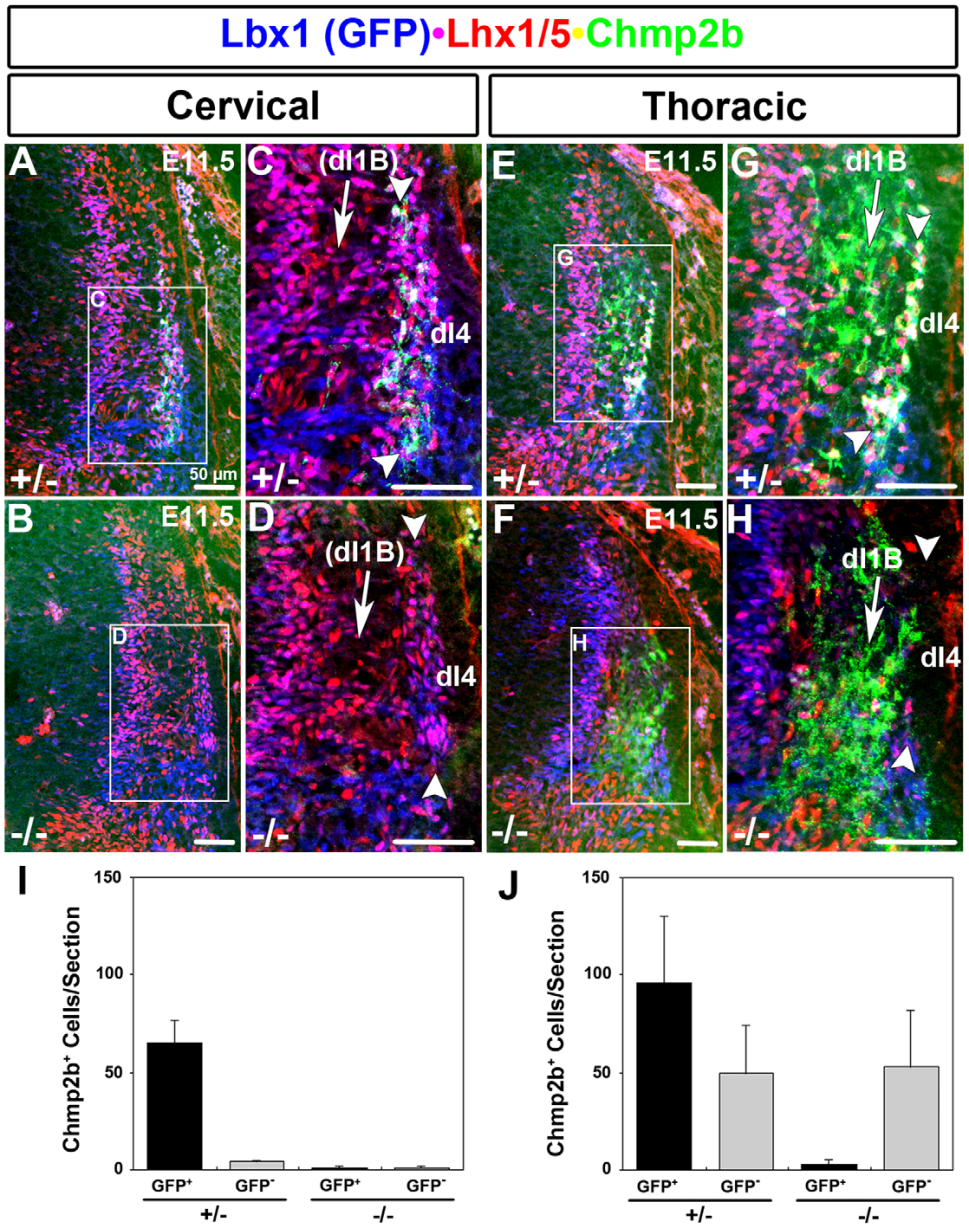
Lbx1-Independent Domain Absent at Cervical Levels. (A–D) Dorsolateral neural tube at cervical levels. A lateral column (bracketed by arrowheads) shows cells that are colabeled by Lbx1(GFP) and Lhx1/5. A subset of these cells are also labeled by Chmp2b in heterozygotes but not in mutants. The size of the lateral column is also reduced, possibly reflecting the loss of dI4 cells, as observed at thoracic levels. The *Lbx1*-independent expression domain in the circumferential trajectory, representing dI1B cells, is absent in both genotypes. (E–H). Dorsolateral neural tube at thoracic levels. A lateral column (bracketed by arrowheads) shows dI4 cells that are colabeled by Lbx1(GFP) and Lhx1/5. Almost all of the cells in this column are labeled by Chmp2b in heterozygotes. The column is absent in mutants. A large *Lbx1*-independent expression domain in the circumferential trajectory, representing dI1B cells, is present in both genotypes. It is adjacent to the dI4 column and obscured the loss of Chmp2b RNA in the dI4 column at thoracic levels (see results). (I, J) Quantification of cells in GFP/Lhx1/5/Chmp2b labeled heterozygote sections at cervical (n = 4) and thoracic (n = 4) levels, respectively. Primary antibodies against GFP, Lhx1/5, and Chmp2b were detected using appropriate Cy2 (green), Cy3 (red), and Cy5 (infrared)secondary antibodies, respectively. Colors were electronically switched, for clarity, to those indicated by the labels.

Chmp2b protein expression at E11.5 was also observed in vertically oriented neurons along the circumferential trajectory followed by commissural interneurons. These neurons are seen as blue-only cells in Fig. 5E, or as green-only cells in Fig. 6 E and G. The dI1, dI2, dI3, dI6, and V0 populations, as well as a subset of the dI5 population, are known to contribute neurons to the circumferential, commissural trajectory. The observed vertically-oriented Chmp2b^+^ neurons lacked Lbx1(GFP) and were therefore not dI5 or dI6 neurons. They were located just medial to the column of Lhx1/5^+^ dI4 association interneurons at E11.5 and lacked the Lhx1/5 expression seen in dI2 neurons (Fig. 5E, arrowheads). Isl1 is expressed in the dI3 population of commissural interneurons. Chmp2b staining was not observed in Isl1^+^ cells along the circumferential trajectory (data not shown). Lmx1b is expressed in both circumferential and lateral dI5 neurons. Chmp2b staining was not observed in Lmx1b^+^ cells (Fig. 5 I, J)). Evx1 is expressed in V0 neurons. Chmp2b expression was not observed in Evx1^+^ neurons (data not shown). Assuming that the known dI2, dI3, and dI5 SSTF markers are not downregulated, the data above eliminate all except the dI1 population as potential sources of this Chmp2b expression domain.

Brn3a is expressed in dI2, dI3, dI5. It is expressed in only a subset of the dI1 population at E11.5 [34]. Chmp2b expression was also not observed in Brn3a^+^ neurons (Fig. 5 K, L; single channel images of Fig. 5K are shown in Fig. S1). Thus, the vertically oriented Chmp2b^+^Lbx1(GFP)^−^ neurons along the circumferential trajectory belong either to the subset of dI1 neurons that lack Brn3a expression (dI1B) or to some as yet undefined population. The dI1B population at E12.5 moves out of the circumferential trajectory and settles at the lateral margin below the sulcus limitans [35], [36]. This is precisely the location of the Lbx1-independent Chmp2b expression domain at E12.5.

### Thoracic-Restricted dI1 Expression of Chmp2b

The laterally-settling dI1B population is present at thoracic but not cervical levels at E12.5 [36]. Chmp2b RNA expression in E11.5 heterozygotes and mutants was compared along the entire body axis. Mutant *s*ections taken at cervical levels showed a complete loss of the lateral column of Chmp2b RNA expression, whereas mutant sections taken at more caudal levels retained a column of Chmp2b expression (data not shown). These data suggested that the dI1-specific *Lbx1*-independent expression domain of Chmp2b was absent at cervical levels but present at thoracic levels. Immunohistochemistry confirmed this hypothesis. A Lbx1(GFP)^+^ Lhx1/5^+^ Chmp2b^+^ column of neurons was observed at the lateral margin of the neural tube at both cervical and thoracic levels (Fig. 6A, C, E, G; single channel images of panels C and G are given in Fig. S2). These triple labeled cells were lost in mutants (Fig. 6 B, D, F, H; single channel images of panels D and H are given in Fig. S2), indicating that they were the dI4 population for which Chmp2b expression is *Lbx1*-dependent.In contrast, Chmp2b^+^Lbx1(GFP)^−^ Lhx1/5^−^ neurons along the circumferential trajectory were observed at thoracic levels but not cervical levels (Fig. 6. A, C, G, E; single channel images of panels C and G are given in Fig. S2). The expression of Chmp2b in these cells was retained in mutants, indicating that it did not depend on *Lbx1.* The quantified results (Fig. 6 I, J) graphically illustrate the presence of an *Lbx1*-independent, GFP^−^ population of Chmp2b^+^ cells that exists at thoracic, but not cervical levels. The *Lbx1*-independent Chmp2b expression domain therefore shares the migration route at E11.5, the settlement zone at E12.5, and anterior-posterior restriction across the cervical-thoracic boundary at E12.5 with the dI1B population. In addition, it lacks markers for all of the other populations known to exist at these locations..

Chmp2b expression in the dI4 column depended on *Lbx1* both at cervical and thoracic levels (Fig. 6). The SSTF code of dI4 neurons is respecified to a dI2-like code in *Lbx1* mutants. This respecification is accompanied by altered migratory behavior. Respecified dI4 neurons move along the circumferential trajectory rather than moving laterally to aggregate into a lateral column. The apparent loss of the Chmp2b^+^ lateral column could therefore be due to relocation of these cells rather than a gene regulatory event. However, it appears that this is not the case. No ectopic Chmp2b^+^Lbx1(GFP)^+^ cells were observed in the circumferential trajectory of mutants. This was particularly evident at cervical levels, where such a population could not be obscured by the *Lbx1*-independent Chmp2b expression of the circumferentially migrating dI1 population (Fig. 6 B, D). The loss of the Chmp2b^+^ column in mutants is therefore due to gene regulation rather than cell relocation.

### Dendritic Deployment of Chmp2b Protein to the Dorsolateral Funiculus

**Figure 7:**
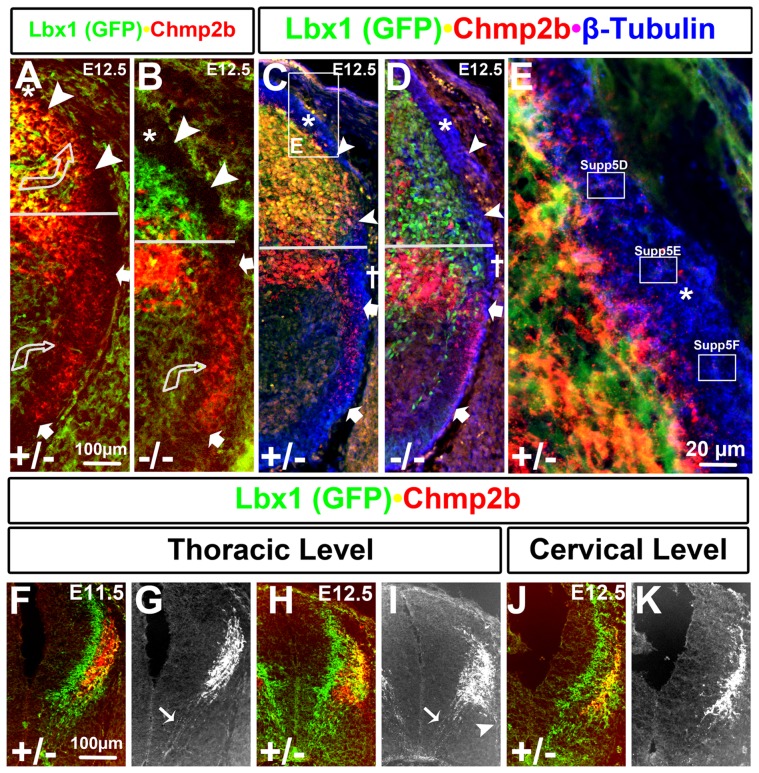
Funicular Localization of Chmp2b Protein. (A) Chmp2b staining between the cellular region and basal lamina was observed in a dorsolateral zone (between arrowheads) and a ventrolateral zone (between arrows)in heterozygotes. (B) The dorsolateral zone is lost in mutants while the ventrolateral zone is retained. (C–E) ß-tubulin Stains longitudinal axons in the lateral white matter as blue puncta. (C) The dorsolateral zone of Chmp2b expression is located between the tubulin stained DLF (*) and lateral funiculus (LF; †) in heterozygotes. It appears smaller in this section than in panel A. (D) Tubulin staining at the sulcus limitans (horizontal line) and in the VLF is severely reduced in mutants. The LF appears to be absent and Chmp2b staining in the VLF has an altered appearance. (E) Chmp2b stained projections enter the heterozygote DLF at E12.5. Afferent projections in this funiculus project longitudinally and have not entered the grey matter at this stage. They are labeled with tubulin (blue). Single channel images of this panel are shown in Fig. S3 A–C. GFP/Chmp2b double labels of the insets are enlarged in Fig. S3D–F. They show coincident labeling of GFP and Chmp2b in the white matter (F–K) The red channel of the color images in F, H, J were converted to greyscale images in G, I, and K, respectively. This allows Chmp2b staining in the circumferential trajectory (arrows) to be seen at thoracic levels at E11.5 (G) and E12.5 (I). No such staining can be seen at cervical levels at E12.5 (K). Note that Chmp2b staining is only seen in GFP stained areas at cervical levels (J), whereas it is observed outside of the GFP stained areas at thoracic levels (F, H).

Chmp2b protein staining in the white matter was first observed at E12.5 (Fig. 2E, Fig. 7). Chmp2b protein is therefore moved into neurite projections during the time when synapses in the mouse neural tube can first be readily observed by electron microscopy [37]. Dorsolateral (between arrowheads) and ventrolateral (between arrows) zones of Chmp2b staining were observed in the white matter of heterozygotes at E12.5 (Fig. 7A). Young E12.5 embryos contain Chmp2b protein at the ventral edge of the dorsolateral funiculus (DLF), which can be labeled by tubulin staining (Fig. 7C), in an area (between arrowheads) that was defined as the association neuropil by early electron microscopy studies of the mouse neural tube [38]. Slightly older E12.5 embryos showed many Chmp2b^+^ projections or puncta within the DLF (Fig. 7A), which were clearly intermingled with the tubulin^+^ axons that course longitudinally along the DLF at this stage (Fig. 7E; single channel images in Fig. S3 A–C).

Chmp2b immunoreactivity in the DLF was lost in mutants (Fig. 7B, D), indicating that it comes from *Lbx1*-dependent sources such as dI4 and/or dI4L^A^ neurons, rather than from *Lbx1*-independent sources such as the dI1B or motor neurons (see below). GFP^+^ cytoplasm in this area can be observed at high magnification, and is tightly associated with Chmp2b^+^ puncta (Fig. S3D–F).

**Figure 8:**
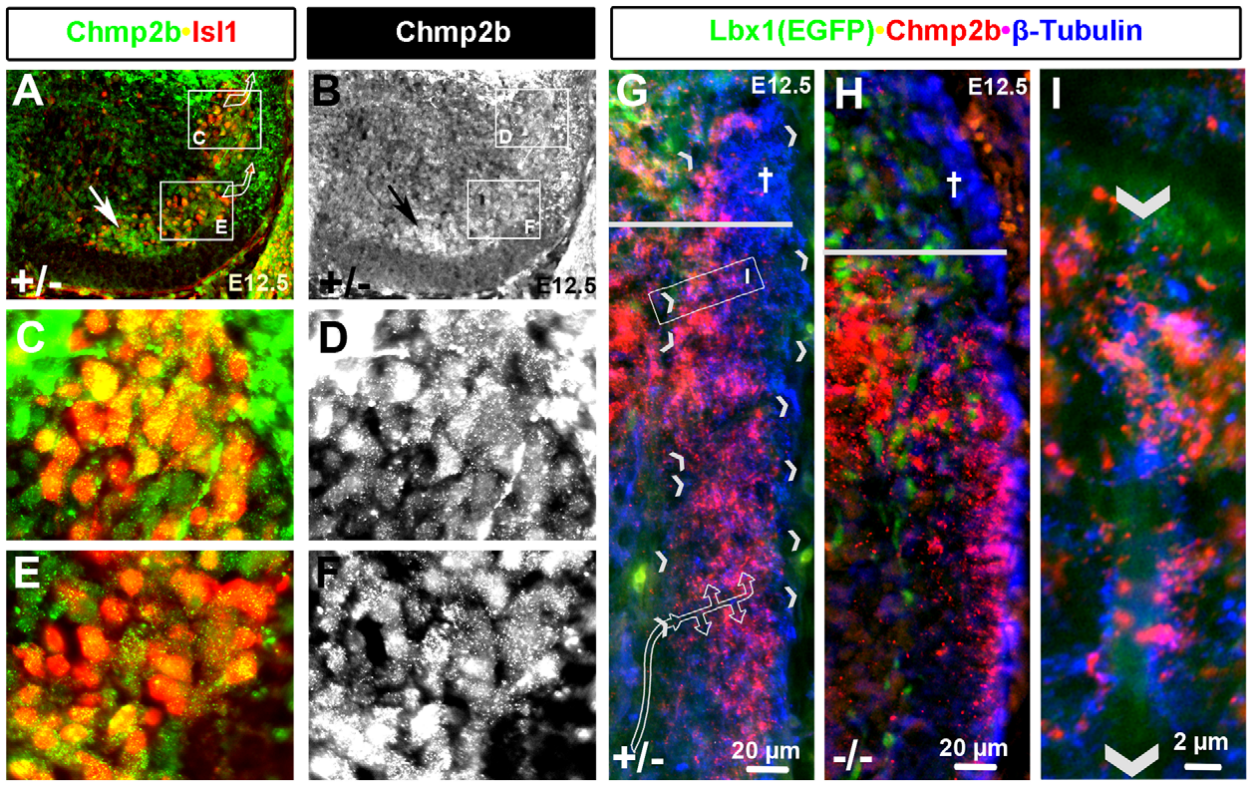
Chmp2b Protein in Soma and Dendrites of Motor Neurons. Chmp2b and Isl1 are co-expressed in motor columns of the ventral horn. (A, C, E) Isl1 primary antibody was detected with Cy3 (red). Chmp2b antibody was detected with Cy5 (infrared) and is shown in green. (B, D, F) Greyscale images of the green channel of images shown in A, C, and E, respectively. Chmp2b staining within soma can be distinguished from staining of projections between soma. The most sensitive secondary antibody (Cy3) and relatively long image acquisition times are required to detect Chmp2b signal in the motor columns. (G, H) Comparison of the VLF of heterozygotes and mutants stained with Chmp2b (red; Cy3) and tubulin (blue; Cy5). Radial breaks in the tubulin stains of heterozygotes (indicated by pairs of arrowheads) may represent the endfeet of radial glial cells (see inset I) that dendrites of motor neurons have been shown to follow into the VLF during early synaptogenesis (diagrammed by flow arrow). Note the loss of tubulin staining in the LF (†) and in the VLF below the sulcus limitans (horizontal line). Chmp2b staining in the VLF is present but distributed differently in mutants. (I) High magnification image of a putative radial glial endfoot that can be seen by background stain in the green channel. Note that Chmp2b and tubulin staining do not colocalize but appear to associate closely on the surface of the endfoot.

**Figure 9:**
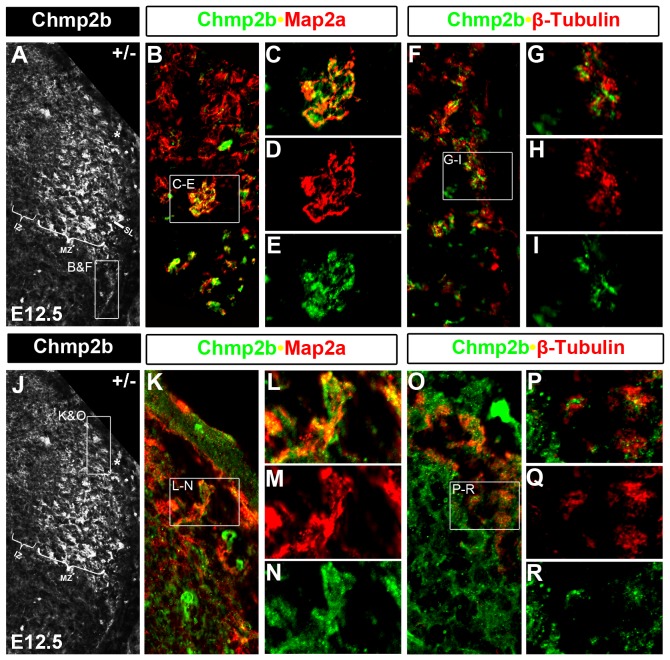
Chmp2b Colocalization with MAP2a in DLF and VLF. (A–I) Comparison of Chmp2b/MAP2a colabeling with Chmp2b/ß-tubulin colabeling in the VLF in adjacent sections (J–R) Comparison of Chmp2b/MAP2a colabeling with Chmp2b/ß-tubulin colabeling in the DLF in adjacent sections. Note that Chmp2b and MAP2a colocalize, while Chmp2b and ß-tubulin are only in close apposition.

Chmp2b^+^ projections in the DLF are unlikely to be afferent axons because Chmp2b RNA or protein were not detected at significant levels in the dorsal root ganglion between E10.5 to E13.5 (data not shown). The Chmp2b^+^ projections in the DLF were also not axons of the dI4 or dI4L^A^ association interneurons because the axons of these neurons are located in the lateral funiculus (LF) and ventrolateral funiculus (VLF) and are redirected toward the floor plate in *Lbx1* mutants [30]. This can be observed here by the loss of tubulin staining from the LF and VLF, but not the DLF, of mutants (Fig. 7C, D and Fig. 8 G, H). The Chmp2b^+^ projections within the DLF are therefore dendrites, rather than axons, of the dI4 and/or dI4L^A^ neurons. MAP2a is classic marker of dendrites, whereas ß-tubulin is typically associated with axons [39]. MAP2a staining of within the DLF coincides closely with Chmp2b staining, whereas tubulin staining only intermingles closely with it (Fig. 9 J–R).

### Dendritic Deployment of Chmp2b Protein in the Ventrolateral Funiculus

The ventrolateral zone (between arrows) of Chmp2b protein staining in the white matter corresponded to the VLF. Staining was of similar intensity in mutants and heterozygotes, indicating that Chmp2b was carried into the VLF from one or more of the neuronal populations that express Chmp2b in an *Lbx1*-independent fashion, either dI1B and/or motor neurons (see below).

Chmp2b immunoreactivity was abnormally localized within the VLF of E12.5 mutants. It was less finely speckled and distributed in later embryos (Fig. 7 A, B), and aggregated along the inside of the basal lamina in earlier embryos (Fig. 8 G, H). The severe loss of longitudinally projecting tubulin^+^ axons in the VLF and LF (dagger) can explain the abnormal localization of the Chmp2b protein in mutants. Far fewer tubulin^+^ puncta were observed in both the VLF and LF of mutants (Fig. 7C, D; Fig. 8G, H). In contrast, tubulin^+^ puncta were not lost from the DLF or ventral funiculus of mutants. The loss of axons from the LF and VLF is due to a re-direction of association axons to the circumferential trajectory in *Lbx1* mutants [30].

High magnification images show that both Chmp2b and tubulin immunoreactivities are punctate within the DLF (Fig. 7E) and VLF (Fig. 8 G, I). Tubulin^+^ puncta mark cross sections of the longitudinally projecting axons. Chmp2b^+^ puncta were closely intermingled, but only rarely overlapped with, the tubulin^+^ puncta of both the DLF and VLF. MAP2a colabels Chmp2b^+^ structures within the VLF, whereas ß-tubulin only intermingles closely with them (Fig. 9 A–I). The Chmp2b expressing neurites in the white matter therefore appear to be dendrites rather than longitudinal axons. Moreover, the punctate nature of the Chmp2b^+^ structures contrasts sharply with the larger, more flowing nature of GFP^+^ cytoplasm (Fig. S3 D–F) in the DLF. Chmp2b may therefore be associated with filipodia or nascent dendritic spines.

### Lbx1-independent Chmp2b Expression in Motor Neurons

Chmp2b protein in dendrites within the VLF was *Lbx1*-independent and should therefore come from the dI1B population rather than the dI4 and/or dI4L^A^ populations. However, electron microscopy studies show that the dendrites of motor neurons make the first synapses of the mouse neural tube within the VLF [40], and we had observed *Lbx1*-independent Chmp2b RNA expression in the motor columns at E12.5 (Fig. 1A, B). Chmp2b protein was not immediately apparent in the motor columns because levels were much lower than in the dI4, dI4L^A^ and dI1B populations (Fig. 1C, D).

High gain exposures with high magnification lenses were used to determine that Chmp2b staining was associated with the cell bodies surrounding Isl1^+^ nuclei at E12.5 (Fig. 8A–F; single channel Isl1 images in Fig. S4) It should be emphasized that Chmp2b immunoreactivity in motor neurons was far weaker than in dI4, dI4L^A^ or dI1B cells. Chmp2b protein could not be detected in the ventral horn at E11.5 using the same high-gain methods (data not shown). Chmp2b RNA was also not ever observed in the ventral horn at E11.5 (Fig. 5A–C; data not shown). Chmp2b gene expression in motor neurons therefore initiates between E11.5 and E12.5.

We could detect Chmp2b protein readily in the dI4, dI4LA, and dI1B populations, but required higher sensitivity to detect it in the in motor neurons. This could be explained in a variety of ways. The Chmp2b RNA could be less efficiently translated or the Chmp2b protein could be more rapidly degraded in motor neurons than in interneurons. More likely, the Chmp2b protein could be moved rapidly into the dendrites of motor neurons as they extend into the VLF.

The dendritic projections of motor neurons at this stage at thoracic levels have been described by electron microscope studies in mouse embryos [38], [40], [41], [42]. These electron microscope studies show that the dendritic growth cones of motor neurons ascend dorsally at the edge of the gray matter until they come into contact with the laterally directed projections of radial glial cells. They turn and crawl laterally along these projections into the ventrolateral funiculus, where they begin to make synapses with longitudinally projecting axons. The collaterals that begin to mature synapses move, or grow away from, the radial glial projection (see arrow in Fig. 8G).

Chmp2b^+^ tubulin^−^ puncta intermingled with Chmp2b^−^tubulin^+^ puncta in the VLF (Fig. 7C, 8G). Both types of puncta were associated with a much larger, laterally-oriented structures that lacked both tubulin and Chmp2b signal, but which could be observed by background signal in the green channel. These structures appear to emerge from the grey matter and span the nascent white matter all the way to the basal lamina. They therefore closely match the morphological descriptions of radial glial end-feet. Paired arrowheads indicate the location of these structures in the VLF (Fig. 8G). An enlargement shows how the two types of intermingled puncta associate with them (Fig. 8I). Radial glia can be labeled by epitopes, such as RC2 or Rat-401 [43] of the neurofilament protein nestin. Nestin also marks the ventricular zone of the neural tube. Chmp2b immunoreactivity does not coincide, but associates closely with nestin immunoreactivity (Fig. S5), indicating that the radial glial endfeet do not express Chmp2b, but come into close apposition with structures that do.

The axons of motor neurons project through the ventral root, where no Chmp2b protein was observed (Fig. 7C). The dendrites of motor neurons in the VLF tend to come from the lateral, rather than the medial, motor columns at this stage. The lateral motor columns showed more intense RNA (Fig. 2; data not shown), but less intense protein (arrow, Fig. 8A), stains than the medial columns. Taken together, the observations are consistent with the idea that protein translated from Chmp2b RNA in motor neurons of the lateral column are rapidly moved into dendrites in the VLF.

## Discussion

### Lbx1 Target Selection Depends on the Precise Molecular State of the Cell

Specification of SSTF combinations in postmitotic neuronal populations is thought to bring about the cell-type specific expression of non-SSTF genes, so that the appropriate collective of catalytic, trafficking, signaling, and structural proteins becomes available to each developmentally-specified population as it matures. Our results indicate that the set of target genes influenced by a homeodomain gene such as *Lbx1* is not only determined by the binding specificity of the SSTF itself, but depends strongly on the cell type in which it is deployed. This has previously been observed for SSTF target genes of *Lbx1*, such as Pax2, Lmx1b, Isl1, and Foxd3. Here we show cell-type specific regulation by *Lbx1* also occurs in a non-SSTF gene, namely Chmp2b.

While temporal and spatial considerations suggest that these five target genes are direct target genes of Lbx1, physical occupancy of CRMs near any of these five target genes by the Lbx1 protein has not yet been demonstrated. Even if these targets are indirectly regulated by an intermediate SSTF, the cell-type specificity of their *Lbx1*-dependent regulation implies that the Lbx1 homeodomain protein does not trigger a standard hierarchical transcriptional cascade wherever it is expressed. If it did, then one would expect to see identical changes in the expression of target genes in all cells where Lbx1 protein is expressed. The set of *Lbx1* target genes is likely to differ in each cell type where the Lbx1 protein is deployed. The fact that *Lbx1* does not equivalently regulate non-SSTF target genes in each cell type where Lbx1 is expressed indicates that *Lbx1* plays different functional roles in each Lbx1^+^ cell type. It is therefore inappropriate to ascribe specific biological, physiological, or neural functions to *Lbx1* without specifying the cell type(s) or population(s) in which it performs these specific functions.

### Population-Specific Regulation

The precise mechanism which produces population-specific Chmp2b expression patterns is likely to remain unresolved until the CRMs in the Chmp2b locus are identified and their occupancy by SSTFs characterized in each neural tube population as a function of developmental time. Clusters of CRMs work together to generate the correct temporal and spatial expression of each gene during embryogenesis of metazoans. The direct interaction between SSTFs and CRMs has been intensively studied in more tractable systems than mammals [44], [45], [46], [47], [48], [49]. One of the paradigms to emerge from these studies is that CRM clusters integrate developmental history with time/space-dependent signaling cues to produce spatial gene expression patterns.

This paradigm provides the most cogent means to explain the observed population-specific expression and *Lbx1*-dependence/independence of Chmp2b expression. It is reasonable to suppose that Lbx1 acts as a patterning SSTF and contributes to the Boolean input of particular patterning CRMs near the Chmp2b gene. In each population where Lbx1 is expressed, it is in the company of a different combination of SSTFs. For example, Lbx1 is co-expressed with Pax2 and Ptf1a, but not with Lmx1b or Brn3a, in dI4L^A^ cells. A patterning CRM near Chmp2b that is in the “block” state could be switched to the “allow” state by assembling a Lbx1/Ptf1a/Pax2 complex on it. This would make the Chmp2b gene available for expression in dI4L^A^, but not dI4L^B^, cells. Conversely, Lbx1 is co-expressed with Lmx1b and Brn3a, but not with Pax2 or Ptf1a, in dI4L^B^ cells. A patterning CRM near Chmp2b that is in the “allow” state could by switched to the “block” state by assembling a Lbx1/Lmx1b/Brn3a complex on it. This would make the Chmp2b gene unavailable for expression in dI4L^B^, but not dI4L^A^, cells. Once the Chmp2b gene is made available for expression by appropriate switching at Boolean CRMs, then it must still be activated by time/space dependent signaling cues. Such cues may exist at the periphery of the neural tube and may account for the strong lateral/weak medial Chmp2b protein signals observed at all stages.

Lbx1 regulates hundreds of other non-SSTFs genes and regulation of these targets is also likely to be population-specific. Future array studies should therefore be performed on progressively more “pure” populations, or cell types, rather than on population mixtures. The effects of genetic perturbations on gene expression and SSTF occupancy in CRM chromatin should be evaluated across the genome in such studies.

### Possible Sources of Cell-Type Specificity

Chmp2b expression depends strongly on *Lbx1* function in the Lbx1-expressing dI4L^A^ cell type but fails to be established in the Lbx1-expressing dI4L^B^ cell type. These two cell types are spatially intermingled from the time they emerge as post-mitotic Lbx1^+^ cells until these observations were made, less than 24 hours later. Consequently, both cell types are subject to an identical signaling milieu and cell-type specific upregulation of Chmp2b is unlikely to arise from differential signaling to the two cell types.

The primary cause of differential Chmp2b activation is likely to be due to intrinsic differences in the two cell types that exist prior to the onset of Lbx1 expression. It has been reported that individual late progenitor (dL) cells can asymmetrically divide, into a dI4LA cell and a dI4LB cell, or into a dI4LA cell and dL progenitor cell [50]. Several SSTFs genes expressed in the dL progenitors (*Gsh1/2, Mash1, Ptf1a*) are known to influence the development of the two cell types that emerge from them, but only the expression of Ptf1a is selectively carried into the dI4/dI4LA cell types as they are born [51], [52], [53]. Differential partitioning of a SSTF, such as Ptf1a, during this division would create differential regulatory paradigms in the two daughter cells. Ptf1a and Lbx1 proteins could then act cooperatively at a CRM to bring about selective expression of Chmp2b in dI4/dI4LA cells.

Several SSTFs begin to be selectively expressed in dI4LA (Lhx1/5, Pax2) or dI4LB (Tlx3, Lmx1b) cells shortly after the final cell division and the onset of Lbx1 protein expression. The maintenance, but not the onset, of expression of these proteins appears to be *Lbx1*-independent [30], [31], [54]. These factors could cooperate with Lbx1 at a CRM near Chmp2b to bring about cell-type specific, yet *Lbx1*-dependent activation of Chmp2b.

### Fewer Lbx1-Targets Per Cell Type

Homeobox proteins function collectively in various combinations, at CRMs, to generate new cellular states from previous cellular states. The sensitivity of *Lbx1* function to cellular state is unlikely to apply merely to its regulation of Chmp2b. One of the pressing issues in analyses of homeobox gene function has long been the identification of target genes that would help explain their biological function. We initially undertook flow sorting/microarray experiments to meet this issue head-on in a mammalian system. One of the surprising outcomes was the sheer number of *Lbx1*-dependent targets, both in the SSTF [29] and non-SSTF (this report) subsets of the data. In our previous reports, we carefully evaluated the issues of significance by a method called fold-scanning and concluded that these many changes are reproducible and do not merely result from measurement noise. In this report, we examine a single non-SSTF target, which showed a large fold-change, and show that it is not uniformly regulated in all Lbx1-expressing cell types. Thus, the hundreds of *Lbx1-*targets observed in pooled Lbx1-expressing cell types (Fig. 1) appear to be an aggregate target set, consisting of many different, partially overlapping, cell-type specific target sets. The current state of knowledge indicates that there are five Lbx1-expressing cell types. However, it is likely that more cell types will be defined as SSTF codes become more refined. The number of *Lbx1*-dependent targets in each cell type is therefore expected to be smaller than initially suggested by array analyses of flow-sorted Lbx1^GFP^ cells.

### Biochemical Role of Chmp2b in Nascent Interneurons

Biochemical studies indicate that the ESCRT proteins play a role in endocytic sorting, recycling, and destruction of monoubiquitinated transmembrane proteins. All cells have transmembrane proteins and need to turn them over. The ESCRT system therefore performs a housekeeping function and all of its essential protein components should be expressed in all cells. Our studies indicate that Chmp2b RNA is selectively expressed at very high levels in three nascent interneuronal populations and at moderate levels in motor neurons. The lack of any Chmp2b expression prior to E11.5 and throughout most of the embryo between E11.5 and E13.5 is incongruous with a housekeeping role.

One explanation is that Chmp2b is expressed in all cells, but at much lower levels than in these few populations. Very sensitive techniques were required to visualize Chmp2b protein in the cell bodies of motor neurons (Fig. 8). Light speckling throughout much of the neural tube was also observed in those assays. This speckling was not due to precipitates in our reagents, because it was not observed in the ventricular zone. Clearly, the dividing cells of the ventricular zone also have a need to turn over transmembrane proteins and can do this without Chmp2b protein.

A second possibility is that Chmp2a and Chmp2b perform redundant functions. The array data showed that all 30 of the other ESCRT RNAs were expressed at identical levels in green and white cells (data not shown), which according to the population partitioning model [25], indicated that they are uniformly expressed in the neural tube. The arrays also showed that none of the other ESCRT genes were *Lbx1*-dependent. Chmp2b expression was therefore not required in the dI4, dI4L^A^, dI1B, and motor neuron populations to make up for a lack of Chmp2a. Moreover, loss of Chmp2b in cultured neurons produces a phenotype, indicating that Chmp2a function is not completely redundant [24].

It is likely that Chmp2a is the Vps2 ortholog and performs the housekeeping tasks, while Chmp2b performs a specialized task in neurons that express it at high levels. Chmp2b is structurally related to Chmp6, Chmp4b, Chmp3, and Chmp2a. All of these proteins appear to function in the budding-out of membranes that is performed by the core ESCRTIII complex during ILV formation in endosomes. Other budding-out events include exosome formation, viral bud formation and cytokineses. Dendritic spines are also budding-out from the membrane and Chmp2b has been implicated in their stabilization [24].

### Lbx1-Independent Chmp2b Protein Expression in the VLF

Chmp2b is the only ESCRT component associated with neurodegenerative disease and appears to be required for dendritic spine potentiation in cultured hippocampal neurons. Chmp2b expression was observed only in the embryonic dI4, dI4L^A^, dI1B, and motor neurons populations. The dI4 and dI4L^A^ populations contribute to the association interneurons of the substantia gelatinosa [30], a structure consisting largely of ipsilaterally projecting, local-circuit interneurons [55]. Both the dI4 [51] and dI4L^A^ [53], [54], [56] populations give rise to inhibitory interneurons. The dI1B population gives rise to spinocerebellar neurons [36]. These are excitatory neurons that project long distances to the cerebellum by both ipsilateral and contralateral trajectories. Motor neurons project axons out of the ventral root and are cholinergic. Clearly, the populations that express Chmp2b do not have a common neurotransmitter phenotype, axonal projection trajectory, or axonal projection distance.

However, Chmp2b protein expression appears to be closely associated with the neuronal populations, projections, and synaptogenic areas that form the first cutaneous reflexes. We observed that Chmp2b protein was transported into dendrites in the DLF and VLF at the time when the spinal cord's first synaptogenesis ramps up in these locations. The Chmp2b^+^ dendrites of the motor and/or dI1B populations entered the ventrolateral funiculus at the time and place when the axons of lateral association interneurons make synapses with the dendrites of motor neurons. The Chmp2b^+^ dendrites of the dI4 and/or dI4L^A^ populations entered the DLF at the time and place where primary afferent axons make the first synapses with the dendrites of lateral association interneurons.

The first two types of synapses in the developing neural tube are thought to create the disynaptic reflex in a retrograde manner. Quantitative electron microscope studies in mouse embryos show that the first synapses form in the lateral marginal zone. Very few synapses can be found at E11, but their density increases 50-fold by E12, and steadily rises thereafter [37]. Synapses are only found in the lateral marginal zones from E11 until E14. Synaptogenesis in the “motor neuropil” was found to slightly precede the “association neuropil” in both rats [57] and mice [38]. In these studies, the “motor neuropil” was defined as the marginal zone dorsolateral to the lateral motor column. This corresponds to the dorsal half of the VLF. The “association neuropil” was defined as the marginal zone dorsolateral to an “association nucleus…exhibiting irregular contours” [38], [57]. This “nucleus” corresponds in appearance and location to a cluster of Lmx1b^+^ dI5 cells that forms at the lateral margin at E12.5 (compare Fig. 10 of [57] with Fig. 3G in [30]). The “association neuropil” is therefore located between the DLF (Bundle of Hiss) and LF and corresponds exactly to the dorsolateral zone of Chmp2b expression in the white matter (Fig. 7). The two zones of Chmp2b protein expression therefore correspond to the two successive zones where synaptogenesis begins in the spinal cord to form the disynaptic reflex.

Axon and dendrite formation on both motor [58] and association [59] neurons was studied by Golgi impregnation in mouse embryos between E9.5 and E11.5. Motor and association neurons begin to project axons as their somata migrate away, and their apical projection are withdrawn, from the ventricular zone during the E9.5 to E11.5 interval. Primary dendrites are formed later, after cells pass through a unipolar stage and reorient dorso-ventrally. The axons of motor and association neurons project ipsilaterally, through the ventral root or longitudinally, along the lateral marginal zone (VLF, LF), almost as soon as they are formed. The VLF contains axons from the V1 [60], V2 [61], [62], and Lbx1-expressing populations [30]. The LF contains axons from one or more Lbx1-expressing populations [30] and from the lateral dI1B population [36]. Golgi impregnations of longitudinal axons in the lateral marginal zone of mouse embryos at E12 show that they are thin, extend no more than two segments, rarely have collaterals, and are connected to the soma of lateral association neurons [63]. These relatively immature axons appear to develop synapses with the newly-formed, Chmp2b^+^ dendritic growth cones or dendrites of motor neurons and/or dI1B association neurons from E12 onward.

### Lbx1-Dependent Chmp2b Protein Expression in the DLF

Chmp2b protein expression in the dI4 and dI4L^A^ populations depends on *Lbx1*, whereas Chmp2b protein in the VLF and LF does not. Dendrites of the dI4 and dI4L^A^ populations therefore contributes little to VLF or LF synpatogenesis described above. Instead their dendrites project into the dorsomedial zone that corresponds to the “association neuropil”, which contains the first synapses between primary afferent axons and dorsally projecting dendrites of association interneurons. A detailed DiI tracing study documents the timing of afferent innervation in mouse embryos [33]. Afferent projections from the dorsal root ganglia begin to gather and extend rostro-caudally in the DLF from E10.5–E12.5. The first afferent neurites enter the dorsal grey matter from this funiculus at E12.5. Lateral association neurons lack dendrites at E10.5, but have primary dendrites that project dorsally at E11.5 [59]. Projections in this direction would reach the lateral “association neuropil” prior to the DLF. Our data indicate that the Chmp2b^+^ dendrites of dI4 and/or dI4L^A^ neurons gradually encroach on the DLF from the “association neuropil” very close to the time when the first afferent axons enter the grey matter.

### Biological Role of Population-Specific Chmp2b Expression

The biochemical literature indicates that Chmp2b is part of the ESCRTIII complex that mechanically executes budding-out of membranes. The medical and cell biology literature indicates that normal Chmp2b is essential for formation of potentiated, mushroom-shaped dendritic spines, rather than filipodia or immature spines. This study indicates that Chmp2b expression is restricted to specific populations of postmitotic, yet immature, neurons by a pattern formation SSTF at precisely the time when these neurons are making the first synapses of the first spinal reflex circuit, the cutaneous disynaptic reflex. The Chmp2b protein is being selectively moved into dendritic projections that enter the white matter to contact either longitudinal ipsilateral axons in the VLF or longitudinal afferent axons in the DLF.

We propose that development uses selective Chmp2b expression as a means to imbue particular populations of neurons with the capability to potentiate immature synapses to mature dendritic spines. This capability would only be realized in those particular neurons that create excitatory synapses in appropriate circuits. In this way a genetically-specified developmental program can reproducibly control which types of newborn neurons will contribute to the boot-up the neural network of the spinal cord in each animal and generation without having to hard wire each particular cell. Interestingly, the Chmp2b null mutant appears to have a coordination phenotype [64].

Similar control by Chmp2b may exist in other areas of active synaptogenesis that involve dendritic spines. Notably, the highest levels of Chmp2b expression in adults are observed in regions of the hippocampus and cerebellum that contain the Purkinje and Pyramidal cells, respectively (Allen Brain Atlas [Internet]. Seattle (WA): Allen Institute for Brain Science. ©2009. Available from: http://mouse.brain-map.org.; [65]). It has not escaped our notice that the underlying principle of limiting an essential protein component of a synapse by gene regulatory mechanisms can be broadly applied to constrain the types of neurons that can engage in that type of synapse at each stage of development. In this way, developmental gene regulatory neworks can constrain synapse-formation to particular populations of neurons without dictating the exact outcome.

## Supporting Information

Figure S1 **Single Channel Images of** **Figure 5I and 5K** **.** (A,B) Red and Green channels of Panel 5I in greyscale. (C, D Red and Green channels of Panel 5K in greyscale.)
(TIF)

Figure S2 **Single Channel Images for Panels C, D, G, and H of** **Figure 6** **.**
(TIF)

Figure S3 **Single Channel Images and Double Labeled Enlargements of** **Figure 7E**.
(TIF)

Figure S4 **Red Channel Image of** **Figure 8a** **, C and E in Greyscale.**
(TIF)

Figure S5 **Close Apposition of Chmp2b with Nestin in VLF.** Nestin marks the endfeet of radial glial cells that traverse the VLF. Chmp2b labeling was observed in close apposition to the Nestin label, but was not colcalized.
(TIF)
