# Supplementary figures and images for: Population-Specific Regulation of Chmp2b by Lbx1 during Onset of Synaptogenesis in Lateral Association Interneurons
Source: PLoS One. 2012 Dec 21;7(12):e48573. doi: 10.1371/journal.pone.0048573 (PMC3528757; doi:10.1371/journal.pone.0048573)

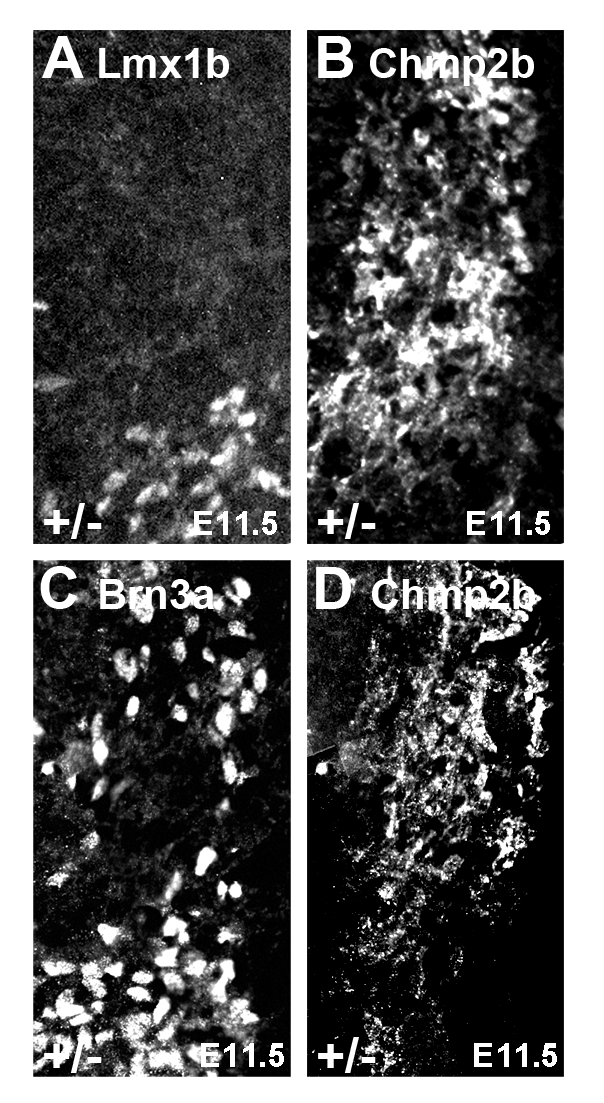

Supplement: Figure S1 — Single Channel Images of Figure 5I and 5K . (A,B) Red and Green channels of Panel 5I in greyscale. (C, D Red and Green channels of Panel 5K in greyscale.) (TIF) [file pone.0048573.s001.tif]

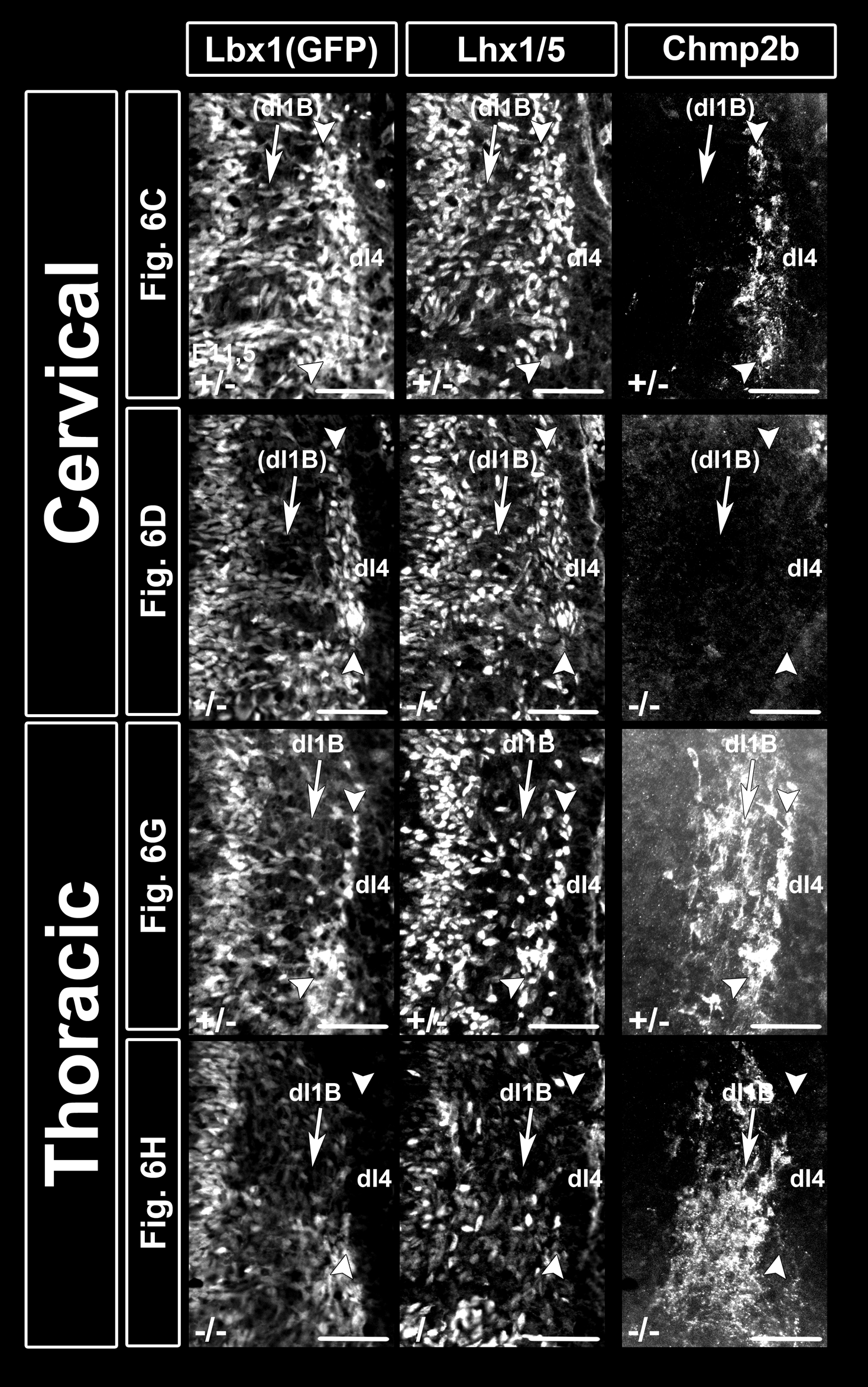

Supplement: Figure S2 — Single Channel Images for Panels C, D, G, and H of Figure 6 . (TIF) [file pone.0048573.s002.tif]

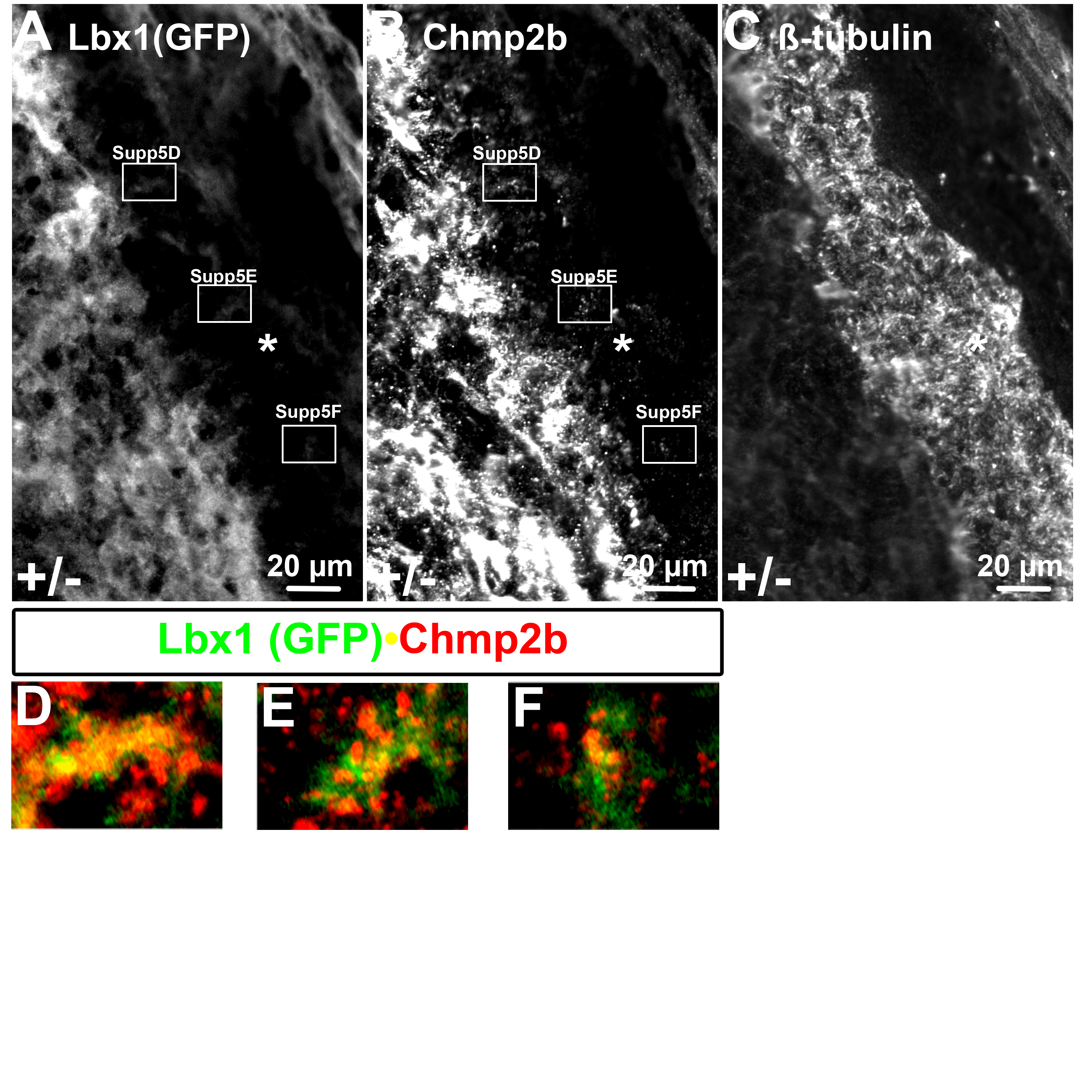

Supplement: Figure S3 — Single Channel Images and Double Labeled Enlargements of Figure 7E . (TIF) [file pone.0048573.s003.tif]

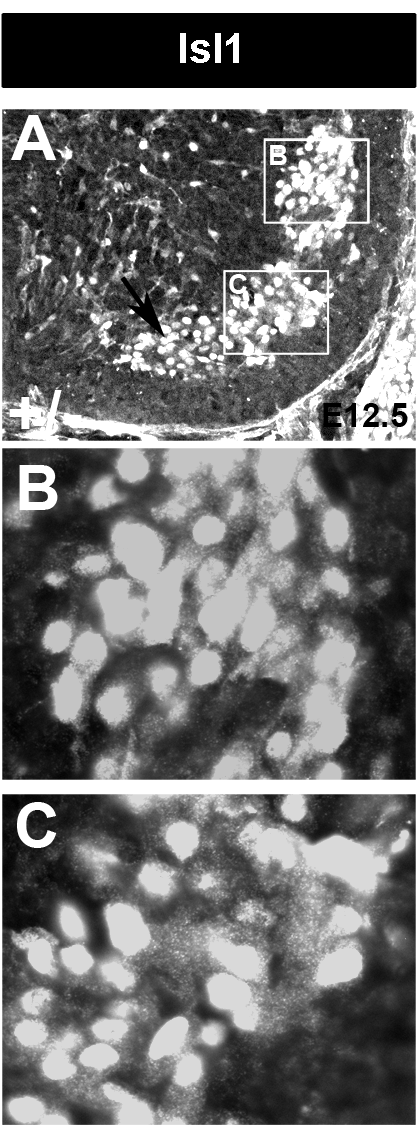

Supplement: Figure S4 — Red Channel Image of Figure 8a , C and E in Greyscale. (TIF) [file pone.0048573.s004.tif]

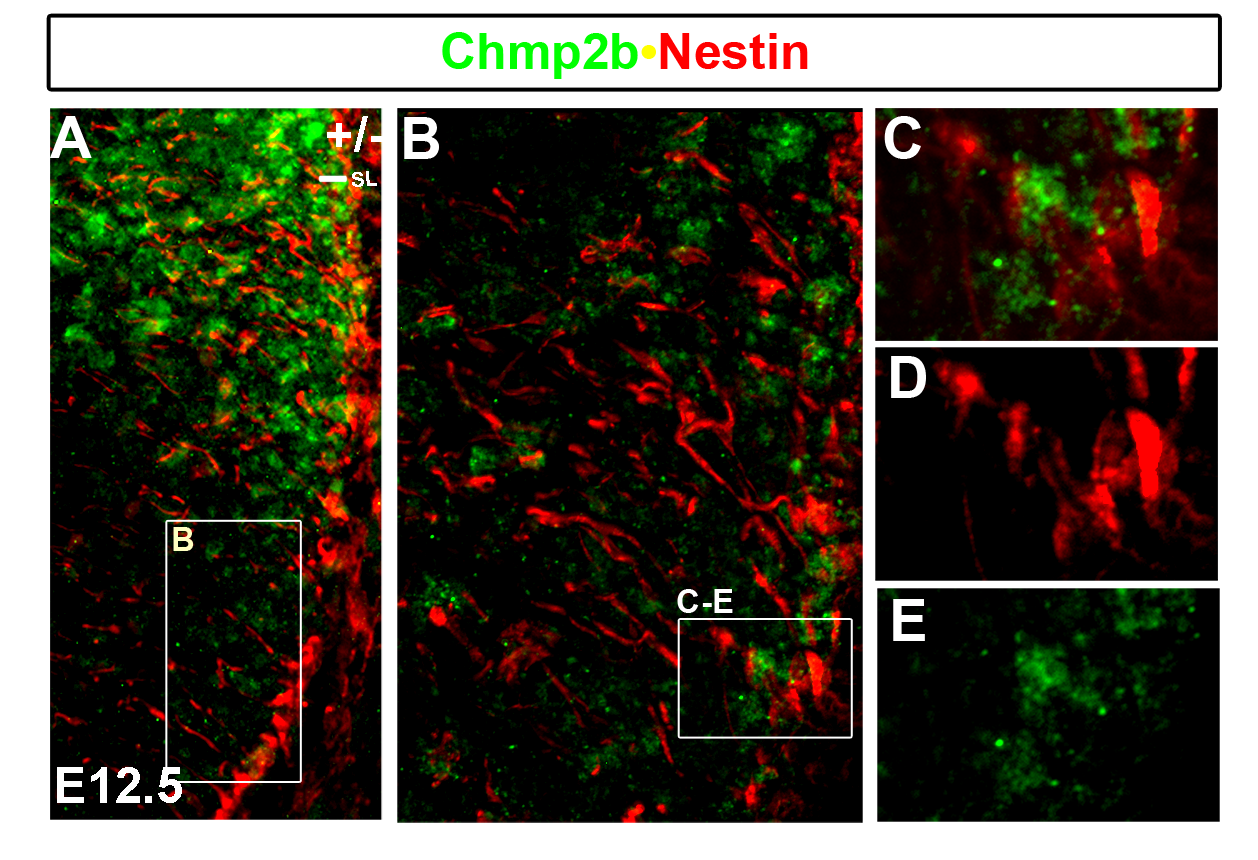

Supplement: Figure S5 — Close Apposition of Chmp2b with Nestin in VLF. Nestin marks the endfeet of radial glial cells that traverse the VLF. Chmp2b labeling was observed in close apposition to the Nestin label, but was not colcalized. (TIF) [file pone.0048573.s005.tif]
